# Innovative Hydroxyapatite–Hydrogel Composites for Cartilage Regeneration

**DOI:** 10.3390/gels12080727

**Published:** 2026-08-15

**Authors:** Anita Ioana Visan, Liviu Duta, Irina Negut

**Affiliations:** National Institute for Laser, Plasma and Radiation Physics, 077125 Magurele, Romania; anita.visan@inflpr.ro

**Keywords:** hydroxyapatite–hydrogel composites, cartilage regeneration, osteochondral repair, responsive biomaterial systems, regenerative medicine

## Abstract

Articular cartilage defects remain a significant clinical challenge due to the limited intrinsic regenerative capacity of cartilage and the inability of many current therapeutic approaches to restore the structure and function of native hyaline tissue. As a result, considerable research efforts have been directed toward the development of tissue-engineering and biomaterial-based strategies capable of promoting more effective regeneration. Among these, hydroxyapatite–hydrogel (HAp–hydrogel) composites have emerged as particularly promising candidates because they combine the biological functionality of HAp with the structural versatility of hydrogel networks. Hydrogels provide a highly hydrated, extracellular matrix-like environment that supports cell survival, facilitates matrix deposition, and enables the localized delivery of therapeutic agents. At the same time, their physicochemical properties can be tailored through a variety of crosslinking approaches and advanced responsive design strategies. The incorporation of HAp, either in nano- or microscale form, contributes to mechanical reinforcement, supports subchondral bone regeneration, and influences cellular behavior through both biochemical and mechanotransductive mechanisms. Beyond promoting chondrogenic differentiation, HAp–hydrogel composites have also been investigated for their capacity to modulate inflammation, stimulate angiogenesis within the subchondral region, provide antibacterial protection, and maintain a microenvironment conducive to tissue repair. This review critically examines recent advances in the development and application of HAp–hydrogel composites for cartilage regeneration, highlighting material design principles, fabrication strategies, healing mechanisms, and the key challenges that continue to influence their clinical translation.

## 1. Introduction

Articular cartilage injuries are prevalent in clinical practice and arise from diverse causes, including trauma, inflammation, aging, and degenerative processes associated with osteoarthritis (OA) [1,2,3]. The limited capacity of articular cartilage for spontaneous repair is closely related to its highly specialized tissue organization. Composed primarily of collagen, proteoglycans, and chondrocytes, the tissue lacks blood vessels, nerves, and lymphatic structures and does not mount the conventional vascular and inflammatory responses that ordinarily contribute to healing after injury [1,4]. Articular cartilage defects may not regain normal structural and functional properties because of the tissue’s limited reparative capacity. Although outcomes can be favorable for small, superficial lesions, the durability of the resulting repair tissue remains a concern, and persistent cartilage degeneration may ultimately impair joint function [5,6]. Because articular cartilage has little intrinsic capacity for regeneration and established treatments often provide incomplete or short-lived repair, current research focuses on tissue-engineering and regenerative approaches. These strategies seek to reproduce the structural and biomechanical properties of native cartilage and may be especially relevant for young, active patients with symptomatic full-thickness lesions [3,7].

Osteochondral defects, which affect both articular cartilage and the underlying subchondral bone, are particularly difficult to repair because regeneration must restore not only the two tissue compartments but also the structurally integrated transition between them [5,8]. Such lesions may result from trauma or degenerative joint changes and, when repair tissue is mechanically inadequate, may contribute to poor stress distribution and further cartilage degeneration [4]. Effective regeneration of the osteochondral unit requires coordinated reconstruction of hyaline cartilage, subchondral bone plate, and underlying vascularized bone. This remains difficult because the two compartments depend on distinct cellular responses, molecular cues, and structural functions [8,9].

Reparative procedures may generate fibrocartilage rather than native hyaline cartilage, and the resulting tissue can deteriorate over time. Because durable cartilage regeneration also depends on restoring appropriate mechanical properties, this limitation remains an important consideration in the design of osteochondral biomaterials [6,10]. In the setting of OA, continued matrix degradation and inflammation can further compromise joint structure, mobility, and overall clinical function [11,12]. In this context, the development of advanced biomaterial scaffolds capable of coordinating the regeneration of articular cartilage and the underlying subchondral bone has become an important focus of osteochondral tissue engineering [8,9].

The development of OA is closely linked to cartilage injury and the limited capacity of articular cartilage for spontaneous repair. As OA progresses, articular cartilage degradation occurs alongside an inflammatory joint microenvironment and pathological remodeling of the subchondral bone. These interconnected changes are associated with pain, reduced mobility, and progressive impairment of joint function [2,13].

The local osteochondral microenvironment is increasingly recognized as an important determinant of regenerative outcome. Therapeutic approaches have therefore begun to combine control of oxidative and catabolic signaling with biomaterial-mediated support for cell survival, matrix deposition, and restoration of tissue homeostasis [13,14]. Given the substantial effects of OA and cartilage injury on mobility, daily activities, and quality of life, considerable research effort has been directed toward regenerative interventions that limit further tissue damage and promote the repair of articular cartilage [15,16].

Depending on lesion characteristics and patient-related factors, cartilage defects may be treated using microfracture, autologous chondrocyte implantation, osteochondral autograft or allograft transplantation, and matrix- or scaffold-assisted procedures, including matrix-induced autologous chondrocyte implantation (MACI) and autologous matrix-induced chondrogenesis (AMIC) [6,7]. Although these approaches have demonstrated clinical benefit in appropriately selected patients, each is associated with distinct limitations that continue to hinder durable cartilage restoration [3,7,10].

Recent advances in cartilage tissue engineering have expanded the range of regenerative approaches, including biomimetic hydrogel scaffolds [1], hydrogel-based 3D-bioprinted constructs [17], and stem-cell-based strategies [18]. These systems are intended to reproduce selected structural, biochemical, and mechanical features of native cartilage rather than its full complexity [17]. Although preclinical studies have shown promising cartilage repair [4], evidence for durable clinical benefit remains limited [18]. Clinical translation is still constrained by several challenges: (i) hydrogel and bioink formulations must balance biocompatibility, mechanical strength, degradation, printability, and support for chondrogenesis [1,17]; (ii) cell sourcing is also problematic because articular chondrocytes are scarce and difficult to expand, whereas (iii) alternative cells may show variable differentiation or hypertrophy [4,17,18]. Additional barriers include integration with host cartilage, standardized and scalable manufacturing, long-term safety and efficacy, and regulatory compliance [17,18].

Hydrogels have emerged as a highly versatile class of biomaterials for cartilage regeneration. Their hydrated three-dimensional (3D) networks can reproduce selected extracellular-matrix and mechanical features of native cartilage while providing a supportive environment for cell encapsulation and matrix formation [1,2]. Depending on their composition and mechanical properties, hydrogel scaffolds may support chondrocyte viability and proliferation, promote cartilage-specific matrix deposition, and enhance sulfated glycosaminoglycan synthesis [1,7,11]. In this context, responsive biomaterial systems have been developed to more effectively modulate interactions with the surrounding biological microenvironment, thereby enhancing their functional performance [19,20].

These materials can be prepared from natural polymers, such as gelatin, chitosan, and hyaluronic acid, or from synthetic polymers, including poly(ethylene glycol) and poly(N-isopropylacrylamide) [1,2]. Chemical modification, crosslinking, and composite formulation allow degradation, porosity, mechanical behavior, and biological activity to be adjusted, although these properties remain closely interdependent [1,7,11,21]. Current designs increasingly incorporate bioactive ligands, anti-inflammatory agents, and controlled-release systems [10,11,21,22]. Stimuli-responsive hydrogels have also been developed to release therapeutic signals in response to temperature, matrix metalloproteinase activity, or mechanical loading within osteoarthritic joints [2,12].

In parallel, hydroxyapatite (HAp), a calcium phosphate bioceramic whose composition closely resembles the mineral phase of bone, has been extensively investigated as a component of scaffolds for bone regeneration and osteochondral tissue engineering due to its biocompatibility and osteoconductive character [23,24]. Nevertheless, its relatively low mechanical strength, pronounced brittleness, and limited functional adaptability restrict its use as a standalone material, particularly in mechanically dynamic or soft tissue environments, where composite approaches are generally favored [11]. Rather than serving solely as an inert filler, HAp can provide a mineralized substrate that supports cell attachment, proliferation, and osteogenic differentiation [25,26]. These properties are particularly useful in osteochondral constructs, where the cartilage layer must be restored together with the mechanically distinct subchondral-bone compartment [27,28]. HAp incorporation may therefore contribute to the mechanical and biological performance of the osseous region, although the extent of reinforcement depends on particle content, dispersion, hydrogel chemistry, and interfacial bonding. As HAp gradually dissolves or remodels, it can provide local calcium- and phosphate-containing cues that influence progenitor-cell behavior, matrix mineralization, and bone formation. These effects are sensitive to ion concentration and release kinetics and should not be interpreted as an inherently beneficial, uncontrolled transition from cartilage to bone [15]. In osteochondral applications, the more appropriate design objective is a spatially confined mineral phase that supports subchondral-bone regeneration without inducing premature mineralization of the overlying cartilage. Gradient and multilayer HAp-containing scaffolds are therefore being explored as means of establishing a more continuous and mechanically stable cartilage–bone interface [27,28].

Within this context, hydroxyapatite–hydrogel (HAp–hydrogel) composites are being explored as osteochondral scaffolds because the two phases address different aspects of the repair environment. The hydrogel phase provides a hydrated microenvironment that supports chondrocyte viability and facilitates ECM deposition, whereas the incorporation of HAp contributes a mineralized phase with greater stiffness and bone-supportive properties [29,30]. The value of these composite systems lies less in creating a chemically uniform scaffold than in distributing materials according to the distinct requirements of cartilage, interface, and subchondral bone. Multiscale printing has been used to combine cell-laden hydrogels with mechanically reinforcing calcium phosphate structures while maintaining a continuous interface between the soft and mineralized regions [30]. More recent gradient hydrogel systems containing doped HAp have likewise been designed to reproduce spatial changes in stiffness and composition across the osteochondral unit and have produced substantial repair in full-thickness osteochondral defects in rats [28]. 3D printing and bioprinting are particularly useful in this setting because they allow control over scaffold geometry, porosity, material distribution, and the placement of biological cues. These capabilities have supported the development of heterogeneous or multilayer constructs in which chondrogenic and osteogenic environments are assigned to separate but interconnected regions [29,31]. Taken together, the available evidence supports HAp–hydrogel composites as promising candidates for osteochondral tissue engineering, particularly when the mineral phase is introduced as a spatial gradient or confined to a bone-oriented compartment.

The present review aims to provide a critical assessment of recent advances (2020–2026) in the design and application of HAp–hydrogel composites for cartilage regeneration. Particular attention is given to material design considerations, fabrication strategies, and the mechanisms underlying cell–material interactions. In addition, key challenges—including mechanical stability, degradation behavior, and translational constraints—are systematically examined. By integrating recent developments across materials science, bioengineering, and regenerative medicine, this review offers a coherent and balanced perspective on the potential of HAp–hydrogel composites to support functional cartilage regeneration. At the same time, it seeks to delineate relevant directions for future research within this rapidly evolving field.

## 2. Conceptual Framework and Literature Selection

Given the broad scope and inherently multidisciplinary character of cartilage regeneration research, particularly in the area of HAp–hydrogel composites, the available literature extends across several interconnected fields, including biomaterials science, tissue engineering, regenerative medicine, pharmaceutical sciences, and biofabrication technologies. Consequently, a comprehensive examination of all published studies would be neither practical nor sufficiently focused within the framework of a single review. To provide a balanced and contemporary perspective, the present work deliberately concentrates on publications appearing between 2020 and 2026. This timeframe was selected to capture the most recent developments in material design, fabrication strategies, biological performance, and translational applications, while ensuring that the discussion reflects the current state of the field and its emerging directions.

Rather than relying exclusively on predefined keyword-based search strategies, a thematic and concept-oriented approach was adopted for the selection and organization of the literature. Although keyword searches represent a valuable tool for identifying relevant publications, their rigid application may inadvertently exclude important interdisciplinary contributions or generate an excessive number of records with limited contextual relevance. For this reason, the review was structured around major research themes and technological developments that have shaped recent advances in HAp–hydrogel systems. Relevant studies were identified through a combination of expert-guided literature exploration, manual screening of high-impact journals accessible through open-access sources or institutional subscriptions, and critical assessment of their alignment with the thematic objectives of each section. This approach was intended to facilitate a coherent and critical synthesis of recent progress while maintaining scientific rigor, transparency, and relevance to the evolving landscape of cartilage regeneration research.

## 3. Hydroxyapatite in Biomedical Applications

### 3.1. Structure and Properties

HAp is a calcium phosphate-based bioceramic with the stoichiometric formula Ca_10_(PO_4_)_6_(OH)_2_ and a Ca/P molar ratio of 1.67. Owing to its close resemblance to bone mineral, HAp is recognized as one of the principal inorganic constituents of human hard tissues [32,]. This material exhibits high chemical stability, low solubility, excellent bioactivity, and remarkable biocompatibility, characteristics that have established its importance in bone tissue engineering and regenerative medicine [32]. In contrast to stoichiometric synthetic HAp, biologically derived HAp frequently presents non-stoichiometric Ca/P ratios and may contain trace ionic substitutions, such as carbonate, magnesium, sodium, or strontium, depending on the biological source and synthesis conditions employed [32,33].

The physicochemical properties of HAp, including crystallinity, are strongly influenced by the synthesis route and precursor source, which subsequently affect its mechanical and biological performance. Increased crystallinity has been associated with delayed bioceramic resorption and dissolution in physiological environments [33]. Consequently, ion-doped HAp systems have attracted increasing attention, as ionic substitutions enable modulation of the HAp structure and functionality, thereby producing materials that more closely replicate the hierarchical organization and functional characteristics of native bone [32,34].

The crystallinity and nanoscale morphology of HAp represent critical factors governing its physicochemical and biological behavior. Nano-HAp, particularly in form characterized by low crystallinity and nanoscale dimensions, is considered highly suitable for mimicking natural bone architecture and promoting osteogenic cellular activity [32,35]. Moreover, nano-HAp/bioactive glass composites have been intensively investigated as bone-regeneration scaffolds because they combine osteoconductivity, bioactivity, and improved mechanical performance. Furthermore, previous studies have emphasized the importance of low crystallinity and nanoscale dimensions in reproducing the structural features of natural bone [35]. Variations in synthesis and processing parameters can substantially influence the physicochemical characteristics of nano-HAp-based materials, including particle morphology, crystallinity, dissolution behavior, and biological response [35,36,37].

Nanostructured HAp is particularly attractive for tissue engineering applications due to its nanoscale dimensions, elevated surface-area-to-volume ratio, and exposed crystal faces, all of which enhance protein adsorption, ion exchange, surface reactivity, and cell–material interactions [38]. In addition, HAp structures with lower crystallinity or higher defect density may exhibit enhanced dissolution kinetics and interfacial activity, thereby facilitating apatite formation and osteoblast-like cell adhesion [37,38].

One of the most significant properties of HAp is its bioactivity, reflected by its osteoconductive behavior and its ability to establish direct bonding with bone tissue without the formation of an intervening fibrous layer [39]. Following implantation, HAp readily adsorbs proteins onto its surface, thereby facilitating chemical interactions between the implant and surrounding tissue and promoting intimate bone integration [39]. The osteoconductive character of HAp provides an appropriate template for new bone formation and supports osteoblast attachment, proliferation, growth, and phenotype expression at the implant interface [39]. Furthermore, HAp possesses ion-exchange capabilities and exhibits excellent osteoconductivity and biocompatibility, features that contribute to its widespread use as a scaffold material for bone-cell attachment, proliferation, and osseous tissue integration [34]. Owing to these properties and its compositional similarity to bone mineral, nanostructured HAp demonstrates exceptional bioactivity and biocompatibility, supporting its application in scaffolds, implant coatings, and drug-delivery systems intended for bone-related biomedical applications [40].

The morphology and crystallinity of HAp nanoparticles are strongly dependent on synthesis temperature, ripening duration, and calcination conditions. Nanostructured HAp-containing scaffolds are of considerable interest for bone tissue engineering because HAp crystals can reproduce the nanoscale dimensions of natural bone mineral, while HA mineralization enhances surface wettability, biocompatibility, osteoconductive behavior, and the capacity to stimulate new bone formation in vivo [36,41]. In parallel, porous HAp scaffolds, including structures with interconnected porosity, have been investigated as platforms for localized drug delivery and bone-regeneration applications, with several formulations demonstrating the ability to support new bone formation following implantation [40]. Recent investigations have shown that HAp mineralization of electroactive carbon nanofiber matrices can improve cytocompatibility, stimulate osteoblast-like cell proliferation under optimized mineralization conditions, and promote substantial new bone formation in vivo [41]. Similarly, hydrogel-based osteochondral constructs incorporating HAp-containing phases can provide mineral-related bioactivity and osteoconductive stimuli, whereas the hydrogel network contributes hydration, softness, and viscoelastic support [28].

The surface chemistry of nano-HAp plays a decisive role in its biological interactions and biomedical functionality. Surfaces enriched in hydroxyl and phosphate groups can interact with proteins, peptides, drugs, growth factors, and other biomolecules through electrostatic interactions and chemical bonding mechanisms [42]. In particular, phosphate, hydroxyl, and calcium-rich surface sites mediate biomolecular interactions and facilitate surface functionalization. Such modifications can alter interfacial properties, including wettability and biomolecular affinity, thereby influencing cell adhesion, proliferation, differentiation, and bone-regeneration-related responses [42,43]. The surface reactivity of HAp can therefore be tailored through chemical functionalization, enabling modulation of its interfacial behavior and biomedical performance [43]. As a result, numerous surface-modification strategies have been developed to enhance bioactivity, antibacterial properties, cell adhesion, and compatibility or dispersion within polymeric matrices [42]. Functionalization of HAp and nano-HAp with amino acids, polysaccharides, proteins, or bioactive peptides has been investigated as an effective approach for improving the bioactivity, antibacterial performance, dispersion stability, and overall functionality of HAp-containing composites intended for biomedical and bone-regeneration applications [42,43].

From a translational perspective, the versatility of HAp arises from the possibility of tailoring its composition, crystal structure, morphology, solubility, surface characteristics, and biological behavior according to specific biomedical requirements [32,34]. Naturally derived ion-containing HAp and surface-engineered nano-HAp systems have been extensively investigated to optimize physicochemical and biological performance. In particular, surface modification of nano-HAp has demonstrated potential for improving bioactivity, antibacterial activity, dispersion within polymeric matrices, and interactions with bone-related cells [33,42]. Furthermore, the incorporation of HAp-based particles into hydrogel matrices has enabled the development of gradient osteochondral scaffolds that integrate mineral-containing phases with hydrated polymeric networks for osteochondral tissue regeneration [28]. Such HAp-containing hydrogel composites are currently being explored for osteochondral repair because they combine HAp-derived mineral cues with biomimetic hydrogel environments and mechanical gradients capable of supporting cartilage and subchondral bone regeneration [28]. Collectively, these structural, physicochemical, and biological characteristics establish HAp as a highly important inorganic biomaterial for bone regeneration, hard-tissue engineering, orthopedic applications, and a broad range of related biomedical uses [32,40].

### 3.2. HA Types

HAp used in biomedical applications is commonly evaluated according to particle size and morphology, chemical composition, ionic substitution, synthesis method, and natural or synthetic origin. These parameters can affect HAp crystallinity, morphology, solubility, bioactivity, mechanical performance, antimicrobial behavior, and interactions with osteogenic cells [32]. The incorporation of HAp into hydrogel-based scaffolds has been extensively investigated as a strategy to modulate the structural, mechanical, and biological properties of regenerative systems designed for cartilage and osteochondral repair [29,44].

Nano-HAp has attracted significant interest because its nanoscale dimensions and apatite chemistry closely resemble the mineral nanocrystals naturally present in bone tissue, which are typically reported to range from approximately 15–100 nm in length, 10–45 nm in width, and 0.5~4 nm in thickness [45]. Compared with larger HAp particles, nano-HAp generally exhibits a higher surface-to-volume ratio and greater surface reactivity, thereby promoting enhanced interactions with surrounding ions, biomolecules, and biological environments [40,45]. Collectively, these features may improve protein adsorption and support cell adhesion, proliferation, osteogenic differentiation, and matrix mineralization in nano-HAp-containing biomaterials. Such effects are commonly attributed to the biomimetic nanoscale characteristics and chemical similarity of nano-HAp to native bone mineral [40,46]. In addition, under appropriate processing conditions, nano-HAp particles can be uniformly dispersed within polymeric hydrogel matrices, enabling the development of composite hydrogels with enhanced physicochemical properties, biomineralization potential, biocompatibility, and osteogenic activity [47].

Despite the advantages associated with nano-HAp, larger HAp particles and HAp-based ceramic structures remain highly relevant for biomedical applications because HAp is generally characterized by chemical stability, low aqueous solubility, biocompatibility, and osteoconductive properties. Recent studies support the continued use of HAp in bone-related implants and scaffold systems, although direct comparisons between micro-HAp and nano-HAp regarding dissolution behavior, surface reactivity, and aggregation remain limited [34]. Furthermore, micro-scale HAp has been investigated as a reinforcing phase in polymeric scaffolds for bone tissue engineering, particularly in applications requiring enhanced mechanical support [48]. In contrast, nano-HAp-containing and nano-HAp-gradient hydrogel systems have been explored for osteochondral regeneration because of their biomimetic composition, improved dispersion with polymeric matrices, and ability to support subchondral bone repair [29,49]. Consequently, the selection of nano- or micro-scale HAp should be tailored to the intended application and to the required balance between biological performance, mechanical support, and material stability.

In recent years, ion-substituted or doped HAp materials have received considerable attention because the incorporation of trace ions naturally present in bone mineral can alter the HAp structure and enhance its biofunctional, osteogenic, and antimicrobial properties [32]. The incorporation of biologically relevant ions into the HAp lattice is widely employed to tailor its physicochemical and biological characteristics. Both cationic and anionic substitutions can influence HAp structure, solubility, osteogenic response, and bone-regeneration capacity [50]. Importantly, ionic dopants not only modify the physicochemical behavior of HAp but may also confer specific biological functionalities, including antibacterial activity, angiogenic stimulation, enhanced osteogenic potential, and, for selected dopants, immunomodulatory effects. Consequently, ion-doped HAp systems are increasingly being investigated as multifunctional materials for bone tissue engineering and regenerative scaffold applications [32].

Overall, ion substitution represents a versatile strategy for extending the biological functionality of HAp beyond conventional osteoconduction. Depending on the substituted ion and its lattice position, doped HAp can exhibit osteogenic, angiogenic, antimicrobial, antioxidant, magnetic, or drug-delivery-related properties, making these materials particularly attractive for multifunctional composite systems in regenerative medicine. Representative examples of ion-substituted HAp systems and their associated biofunctional roles are summarized in Table 1.

Strontium-doped HAp has been extensively investigated because Sr ions can promote osteogenic activity, particularly osteoblast differentiation, while inhibiting osteoclast proliferation, differentiation, and bone resorption. This dual regulatory effect on bone formation and resorption makes Sr-doped HAp particularly attractive for bone-regeneration strategies, especially in applications associated with osteoporosis or impaired bone-healing conditions [51]. Although Sr incorporation can favor osteogenic differentiation and restrain osteoclast-mediated resorption, its effect is not unlimited, and the available evidence supports a composition- and release-dependent therapeutic window rather than a single universally applicable threshold. Low-to-moderate substitution levels generally retain the apatite structure and provide sustained local Sr^2+^ concentrations that support osteoblast viability and maturation; for example, Sr-substituted HAp containing approximately 1–4 at.% Sr preserved the HAp lattice and promoted the maturation of primary human osteoblasts [61], while Sr-containing nanofibrous matrices also enhanced osteoblast proliferation and osteogenic marker expression without detectable cytotoxicity [62]. Nevertheless, increasing Sr replacement progressively expands the unit cell because Sr^2+^ is larger than Ca^2+^, while altering crystallinity, crystal dimensions, solubility, and ion-release kinetics [63,64]. These changes do not necessarily amount to abrupt lattice collapse, but excessive substitution may increase structural disorder, dissolution, and local Sr^2+^ exposure sufficiently to impair mineralization or cellular activity. Recent evidence indicates that the biological response is distinctly dose-dependent: comparatively low Sr concentrations can stimulate osteogenic activity, whereas higher concentrations may suppress matrix calcification, and a systematic assessment of Sr-doped calcium phosphates associated substitution levels of approximately 10–20% with a greater likelihood of cytotoxic responses than levels below 5% [64]. At the same time, the optimal nominal content remains formulation-specific, since a recent concentration-screening study identified 20 mol.% Sr-HAp as the most effective composition within that particular nanoparticle system, despite evaluating substitution levels up to 100% [65]. Importantly, the biological activity of Sr-substituted HAp is governed by the concentration and temporal availability of released Sr^2+^, rather than simply by the nominal amount incorporated into the solid phase. Sr^2+^ release has been characterized in representative HAp-based systems, although the available data do not yet support a single kinetic profile that can be applied to all Sr-HAp–hydrogel composites. In Sr/Zn-co-substituted nano-HAp incorporated into PLGA scaffolds, ICP-MS measurements showed that Sr^2+^ was detectable from the first day of immersion in simulated body fluid, reached its maximum release around day 7, and subsequently declined to a comparatively stable level that remained measurable after three weeks; as expected, the scaffold containing the highest dopant concentration produced the greatest release [66]. A subsequent study of the same material platform confirmed release over 28 days and reported a peak Sr^2+^ concentration of approximately 9.02 µg dL^−1^ for the scaffold containing 4 mol.% Sr/Zn, while also showing that the released amount increased with the nominal substitution level [66]. These findings indicate that bulk Sr content influences ion availability but does not define it independently, since the release profile is also controlled by HAp crystallinity and solubility, particle dimensions, porosity, exposed surface area, polymer degradation, fluid composition, and possible dissolution–reprecipitation processes. Recent analyses of Sr-substituted calcium phosphates similarly emphasize that Sr incorporation within the crystal lattice generally provides more gradual release than surface adsorption or highly soluble Sr-containing phases, but that the resulting kinetics remain strongly dependent on the material formulation and testing conditions [67].

Magnesium-doped HAp has demonstrated enhanced biological performance because Mg plays an essential role in bone development, mineralization, and regeneration. The incorporation of Mg can introduce lattice distortion, reduce crystallite dimensions and potentially increase dissolution susceptibility [47]. In a recent Mg-nHA/PVA/chitosan study [47], the authors compared hydrogels containing 1, 5, and 10% of a pre-prepared Mg-nano-HAp phase rather than HAp samples with systematically varied lattice substitution. Although gradual Mg^2+^ release was demonstrated and the release profile changed with Mg-nano-HAp loading and hydrogel pore structure, neither the crystallinity of each Mg substitution level nor hydrogel mass loss or HAp dissolution in physiological medium was quantified. The extent to which Mg substitution reduces HAp crystallinity cannot currently be expressed as a universal decrease per unit of Mg^2+^ incorporated, because the reported structural response depends strongly on the synthesis route, thermal history, actual lattice occupancy, and method used to calculate crystallinity. In a systematic study of Ca_10−x_Mg_x_(PO_4_)_6_(OH)_2_, with x ranging from 0 to 2, Bystrov et al. [68] observed a progressive reduction in crystallite size from 24.3 nm for undoped HAp to 21.8, 19.8, 18.2, and 13.7 nm at x = 0.25, 0.5, 1.0, and 1.5, respectively, followed by a modest increase to 15.5 nm at x = 2.0. Thus, even within a single material series, the relationship was not strictly linear and should not be interpreted as a fixed loss of crystallinity for each molar increment of Mg. The same study also showed contraction and distortion of the apatite unit cell with increasing substitution, but did not measure degradation under physiological conditions; consequently, it did not establish a direct quantitative correlation between crystallinity and degradation rate [68], which could be seen as a limitation of the study. Moreover, the biological advantages reported for Mg-substituted HAp have not been demonstrated uniformly across all cell types or Mg substitution levels, and therefore they should be interpreted within the context of the individual experimental model. In the study by Zhang et al. [47], the biological characterization focused on biocompatibility, rat bone marrow mesenchymal stem cells (rBMSCs) proliferation, osteogenic differentiation, in vitro biomineralization, and Mg^2+^ release from Mg-nano-HAp/PVA/chitosan hydrogels. In contrast, specific aspects of the initial cell–material interface, including cell adhesion, spreading, and focal adhesion formation, were not examined using dedicated experimental assays. Among the investigated formulations, the 5% Mg-nano-HAp/PVA/chitosan hydrogel exhibited the most favorable biomineralization performance, while the composite hydrogels generally promoted rBMSC proliferation, osteogenic differentiation, gradual Mg^2+^ release, and good biocompatibility. It is important to mention that further increasing the Mg-nano-HAp content to 10% did not provide additional biological benefit [47]. Consequently, the currently available evidence supports the conclusion that Mg-substituted HAp enhances the behavior of mesenchymal stem cells primarily by stimulating proliferation and osteogenic differentiation, but it does not establish a universal Mg substitution level at which these effects are maximized. Instead, the optimal Mg content remains formulation-specific and should be determined experimentally for each HAp-based composite system.

Zinc-doped HAp has also attracted considerable attention because Zn incorporation can enhance both the biological and antibacterial properties of HAp. In particular, Zn-doped HAp has been associated with improved stem-cell proliferation and differentiation, increased osteoblastic activity, and enhanced antibacterial performance. These characteristics make it relevant for coatings, composite systems, and tissue-engineering scaffolds intended to support bone repair while reducing the risk of infection [69]. It is important to mention that the antibacterial and cytocompatibility data available for Zn-substituted HAp indicate that a compatible concentration window can be achieved, although it is specific to the formulation, Zn^2+^-release profile, bacterial strain, and cell model rather than being defined by a universal bulk-doping threshold. A particularly relevant example was reported for a chitosan/agarose scaffold containing Zn-doped nano-HAp at a Zn loading of only 0.03 mol per mol of HAp, corresponding to ~0.2 wt.% Zn; this formulation released Zn^2+^ gradually, remained non-toxic toward eukaryotic cells, and produced bactericidal activity against *S. epidermidis* and *E. coli* (>99.9% killing), together with 98.5% killing of *S. aureus* and bacteriostatic activity against *P. aeruginosa* [70]. Compatibility has also been demonstrated at higher nominal substitution levels under carefully controlled exposure conditions: Zn-HAp nanoparticles containing 14.7% Zn/[Ca + Zn] supported osteoblast proliferation while retaining antibacterial activity when tested at 0.1 mg mL^−1^, illustrating that particle dose and ion availability may be as important as the substitution percentage itself [71]. Similarly, Sr/Zn-co-doped nano-HAp–PLGA scaffolds containing up to 4% dopant achieved 99.7% inhibition of *S. aureus* while supporting primary rat osteoblast proliferation; however, the authors deliberately limited the dopant concentration to 4% because greater Zn^2+^ release could exceed the cytocompatible range [66]. Taken together, these findings show that antibacterial Zn^2+^ release does not inevitably compromise mammalian-cell viability, provided that Zn incorporation and matrix degradation produce a gradual, locally controlled release rather than a rapid accumulation of free ions. Accordingly, Zn-doped HAp–hydrogel systems should not be judged solely by nominal Zn content: time-resolved Zn^2+^ release, bacterial reduction, and cell viability should be measured in parallel, as the concentration that is both antibacterial and cytocompatible will depend on scaffold composition, surface area, crystallinity, degradation rate, and the duration of exposure.

Silver-containing HAp systems have been widely explored for antimicrobial applications because Ag species exhibit activity against both Gram-positive and Gram-negative microorganisms. Ag-doped HAp and HAp-based composites have therefore been investigated as strategies to reduce microbial colonization and biofilm formation on biomedical surfaces. However, the Ag concentration must be carefully controlled to maintain acceptable biocompatibility [72].

Silicon-substituted HAp represents another important class of bioactive materials because Si incorporation can alter HAp surface properties and enhance cellular responses relevant to bone tissue engineering. Recent studies have shown that Si-HAp can promote osteoblast attachment and vessel-like structure formation in osteoblast/endothelial-cell coculture models, highlighting its potential for both osteogenic and angiogenic applications [73].

Collectively, these ion-substituted HAp systems demonstrate how controlled compositional modifications can transform HAp from a passive osteoconductive ceramic into a multifunctional therapeutic biomaterial.

Another important classification distinguishes HAp derived from natural or biogenic sources from chemically synthesized HAp. Synthetic HAp can be produced using methods such as sol–gel processing, chemical precipitation, hydrothermal synthesis, and solid-state reactions, with the selected synthesis route and processing conditions strongly influencing stoichiometry, morphology, grain size, and crystallinity [33]. In contrast, biogenic HAp is generally obtained from natural resources including bovine bone, fish bone, eggshells, and marine-derived structures [74,75,76]. Biogenic HAp often frequently contains endogenous ionic substitutions, such as Mg^2+^, Sr^2+^, Na^+^, and carbonate ions, resulting in a composition more closely resembling native bone mineral. These compositional features may improve osteoconductivity, osteointegration, and remodeling kinetics compared with highly stoichiometric synthetic HAp, although the final performance remains dependent on the source material and processing conditions [77,78].

The biological behavior of HAp is strongly influenced by its origin, processing conditions, crystallinity, and trace-ion composition. Naturally derived or biomimetic HAp may contain biologically relevant ionic substitutions and often exhibits compositional characteristics closer to those of native bone mineral, which can affect dissolution behavior, apatite-forming ability, surface bioactivity, and cellular responses. These properties support its application in bone-regeneration strategies, although the effects on protein adsorption and osteogenic signaling should be evaluated for each specific material system [77]. Nevertheless, chemically synthesized HAp offers important advantages in terms of purity and control over physicochemical properties, including stoichiometry, morphology, and crystallinity. Such characteristics make synthetic HAp particularly suitable for standardized biomedical applications, although scalability and reproducibility remain dependent on the selected synthesis method and process control [33].

Consequently, current research increasingly focuses on combining the advantages of both approaches through biomimetic synthesis strategies capable of reproducing the ionic complexity and nanoscale organization of natural apatite while preserving the processing control associated with synthetic systems. These developments are particularly relevant for HAp–hydrogel composites intended for cartilage and osteochondral regeneration, where precise regulation of mineral composition and biological signaling is essential for achieving functional tissue integration.

### 3.3. Biological Functions of HAp in Healing

HAp is widely employed in tissue engineering because of its biocompatibility, osteoconductive behavior, and capacity for biological functionalization. In HAp–hydrogel composites designed for cartilage regeneration, recent studies have also reported additional roles of HAp, including osteoinductive effects, modulation of angiogenesis, and antibacterial activity, largely enabled by advances in material design and ion-doping strategies. These multifunctional properties support the potential of HAp-based hydrogel systems for cartilage repair, osteochondral regeneration, guided tissue engineering, and implantable biomedical applications.

#### 3.3.1. Osteoconduction

HAp is widely recognized as an osteoconductive material for bone repair because it provides a suitable surface for osteoblast attachment, proliferation, and differentiation, all of which are essential for new bone formation [79,80]. HAp-based scaffolds, commonly produced by freeze-drying or 3D printing, possess a porous architecture that resembles the extracellular matrix and promotes nutrient transport and cell infiltration through interconnected pores [81,82].

Previous studies have shown that the incorporation of mesoporous SiO_2_-HAp particles into chitosan/HAp scaffolds improves pore uniformity, reduces pore size, and enhances compressive strength, thereby contributing to improved osteoconductivity and mechanical stability [79]. Composite systems based on HAp and biopolymers, including chitosan and polycaprolactone, show improved cell compatibility and mechanical performance. These materials support osteoblast growth on mineral-rich regions, while fibroblasts preferentially adhere to the bioactive surfaces [83].

In animal models of rotator cuff tendon-to-bone healing, dual cross-linked COL1/HAp-loaded human amniotic mesenchymal stem cells enhanced collagen fiber alignment, promoted fibrocartilage formation, and increased bone regeneration, highlighting the osteoconductive role of HAp in tissue engineering applications [84]. In addition, injectable HAp/collagen pastes accelerated tendon-bone interface healing in canine models by providing a biomimetic environment that supported osteogenic cell proliferation and extracellular matrix deposition [85].

HAp-modified polyurethane foam scaffolds with a homogeneous HAp distribution have been shown to improve bone repair in calvarial defect models, as confirmed by microcomputed tomography and in vivo analyses, emphasizing the osteoconductive contribution of HAp [86]. Their mineral composition closely resembles that of native bone, which favors protein adsorption and cell attachment [80]. In guided bone regeneration, asymmetric bilayer membranes composed of HAp and chitosan demonstrated both osteogenic and fibrogenic functions. Experiments using rat skull defects revealed that these membranes provided mechanical support for bone regeneration while also exhibiting antibacterial activity that protected the surrounding soft tissue [83].

Recent reviews have highlighted the importance of HAp coatings for metallic implants due to their ability to promote osteointegration and improve corrosion resistance, while also serving as an environmentally friendly and non-toxic alternative to conventional coatings [87]. The osteoconductive properties of HAp can be further improved through surface engineering approaches and the incorporation of nanocomposites, including carbon allotropes and biopolymers, which contribute to the development of smart self-healing systems for long-term biomedical implants [87]. Clinical studies in dogs with comminuted long bone fractures treated using HAp bone grafts combined with collagen membranes reported enhanced bone healing and fewer complications, supporting the role of HAp in osteoconduction during guided bone regeneration [88].

**Figure 1:**
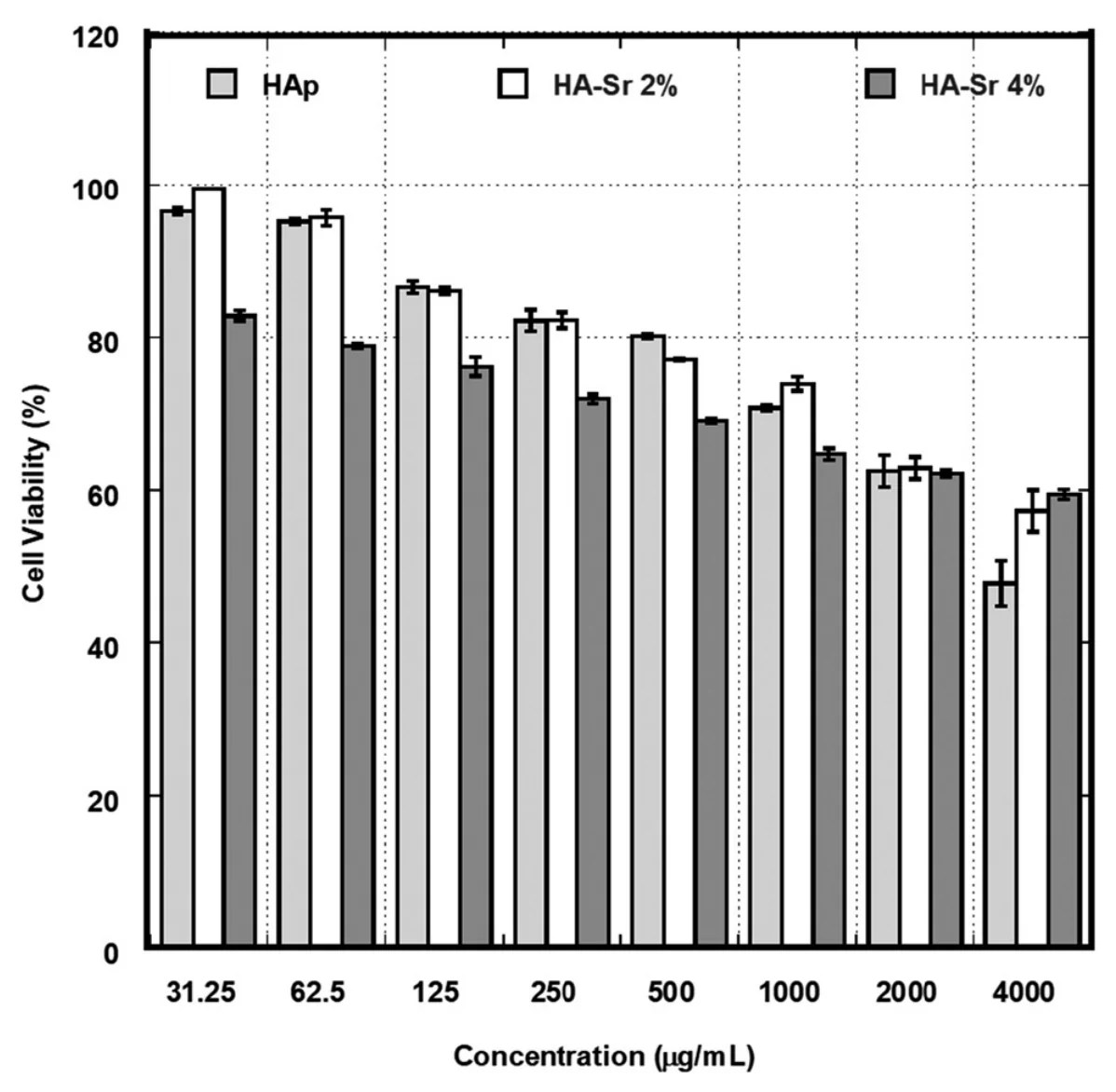
Percentage of viable cells at different concentration ranging from 31.25 to 4000 μg/mL. Reproduced with permission from [80].

Comparative studies of pure HAp and ion-doped formulations have shown that Sr^2+^ incorporation improves bone regeneration and cell viability (Figure 1) while preserving a mineral composition comparable to that of native bone, thereby maintaining the osteoconductive function of the material [80].

**Figure 2:**
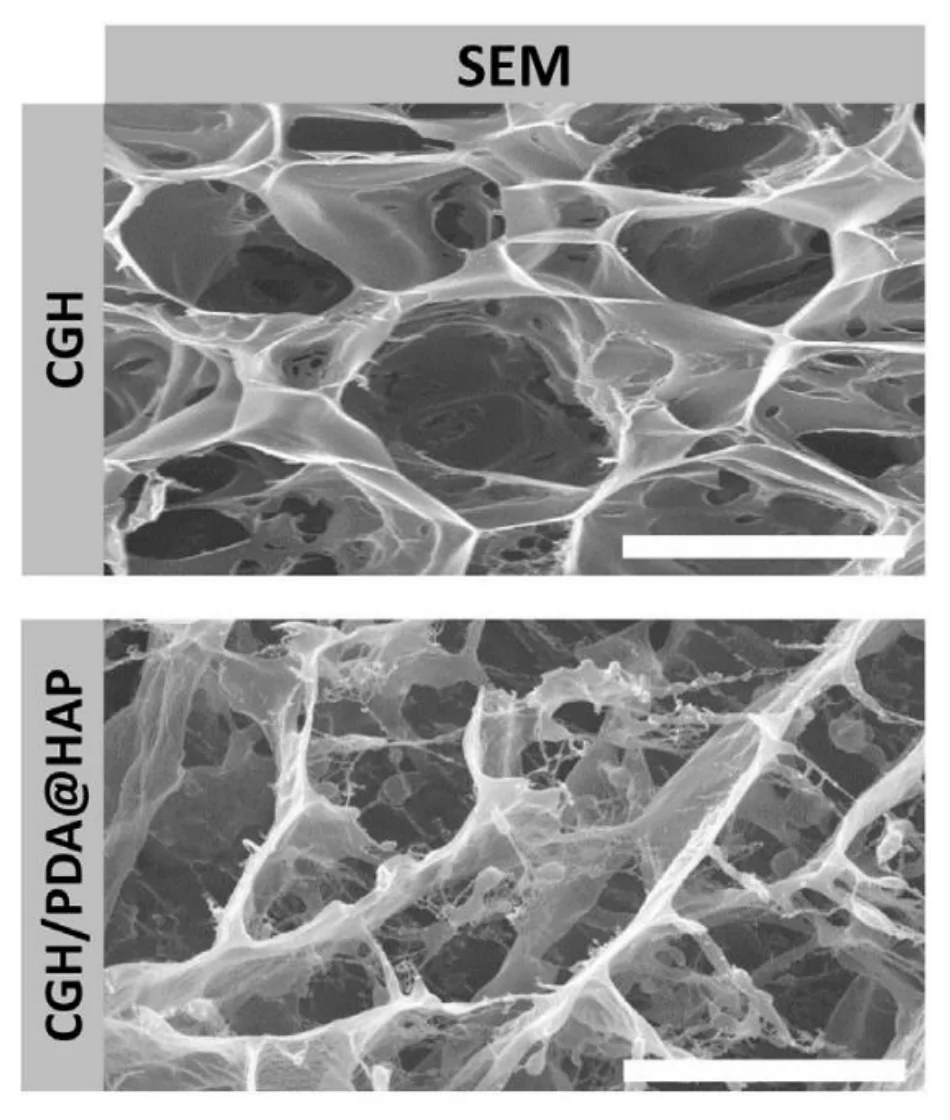
Scanning electron microscopy images of composite hydrogel (chitosan, gallic acid, and hyaluronic acid—CGH) and HAp modified with polydopamine and introduced to CGH (CGH/PDA@HAp) hydrogel. Scale bar: 2.5 μm. Partly reproduced with permission from [89].

In addition, composite hydrogels containing HAp, such as gallic acid-grafted chitosan hydrogels combined with polydopamine-modified HAp, form a three-dimensional porous network (Figure 2) that supports bone marrow stem cell differentiation and stimulates new trabecular bone formation at defect sites [89].

Biphasic composites based on sodium-substituted HAp and diopside pellets have also demonstrated substantial apatite formation in simulated body fluid, confirming their bioactivity and their potential to support osteoconduction in hard tissue engineering applications [90].

#### 3.3.2. Osteoinduction

The osteoinductive properties of HAp have been increasingly investigated through ion doping strategies designed to stimulate progenitor cell differentiation into osteoblasts and promote new bone formation in both in vitro and in vivo models [91]. Among these materials, sodium-substituted HAp composite pellets, particularly the DNA-1 formulation, exhibited elevated alkaline phosphatase (ALP) activity and improved pre-osteoblast viability, indicating enhanced osteoinductive potential while maintaining a favorable balance between bioactivity and mechanical stability [90]. Similarly, strontium-substituted biogenic HAp derived from snail shells showed increased lattice constants, reduced crystallinity, and enhanced cell viability, suggesting that Sr incorporation can improve both the biological response and the suitability of biogenic HAp for bone-related applications [80].

Fluorine and Se co-doping in HAp coatings has also been associated with increased osteogenic activity in osteoblasts together with antiproliferative effects against osteosarcoma cells, demonstrating the potential of ion-substituted HAp coatings for bone repair and dental implant applications [92].

**Figure 3:**
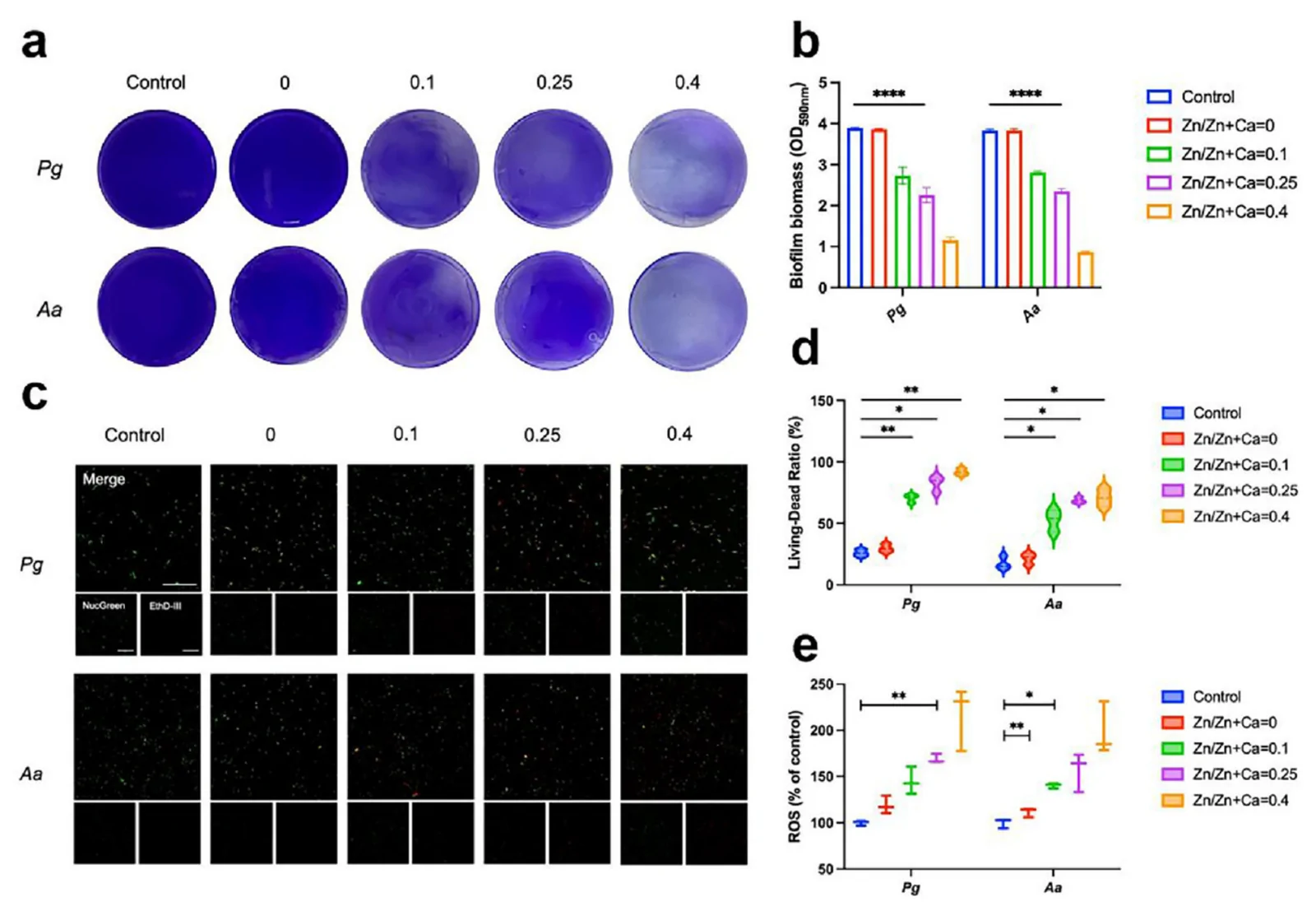
Evaluation of the antibacterial efficacy of Zn-HAp NPs: (**a**) representative crystal violet staining of biofilms, (**b**) quantitative inhibition of biofilm biomass, (**c**) live/dead fluorescent staining of bacteria (green: live; red: dead), (**d**) quantification of bacterial viability from (**c**), and (**e**) intracellular ROS levels induced by NPs. Data are mean ± SD (*n* = 3); * *p* < 0.05, ** *p* < 0.01, **** *p* < 0.0001. Reproduced with permission from [93].

In parallel, Zn-doped HAp nanorods synthesized through one-step in situ mineralization promoted osteogenic differentiation in vitro while providing antibacterial functionality (Figure 3), supporting their use in alveolar bone regeneration [93].

Comprehensive reviews further indicate that metallic ion incorporation, particularly with Sr^2+^, Zn^2+^, and Cu^2+^, can improve the structural and biological performance of HAp by enhancing osteoblast proliferation and bone regeneration [91]. Composite scaffolds based on HAp have also shown promising osteoinductive behavior. HAp-TiC-Ag nanocomposites exhibited favorable mechanical properties and low cytotoxicity, supporting osteoblast proliferation while suppressing cancer cell growth, thereby extending their potential use to both bone regeneration and anticancer applications [94]. In another study, dual cross-linked gradient COL1/HAp scaffolds loaded with human amniotic mesenchymal stem cells promoted tendon-bone interface healing by enhancing fibrocartilage and bone formation, with biomechanical performance significantly higher than that of the control group [84].

In vitro studies have additionally shown that ion-doped HAp can improve tissue engineering performance, osteoconductive behavior, and drug-loading capacity depending on the selected dopant, highlighting its potential for advanced biomedical applications [95].

Polyurethane scaffolds modified with HAp demonstrated enhanced protein adsorption and improved bone repair in animal models, while micro-CT analyses confirmed increased bone regeneration at defect sites [86]. Chitosan/HAp scaffolds have also attracted attention because they combine the biocompatibility of chitosan with the osteoconductive properties of HAp, and recent studies suggest that machine learning approaches may help optimize scaffold design and preclinical performance [81]. Injectable HAp/collagen pastes further accelerated tendon-bone interface healing by providing a biomimetic environment that supported early cell attachment, proliferation, and osteogenic activity [85].

Recent reviews on HAp-based smart self-healing coatings emphasize their combined corrosion resistance, biocompatibility, and osteoinductive potential, making them attractive candidates for next-generation implant coatings with improved durability and patient safety [87]. Clinical studies using HAp bone grafts combined with collagen membranes for the treatment of comminuted fractures also reported favorable healing outcomes, further supporting the osteoinductive role of HAp in bone regeneration applications [88].

#### 3.3.3. Angiogenesis Modulation

The modulation of angiogenesis plays a central role in bone healing because vascularization supports nutrient transport, cell migration, and tissue integration at the defect site [96]. The incorporation of pro-angiogenic agents such as deferoxamine (DFO) into HAp scaffolds, particularly through gelatin methacryloyl hydrogel coatings, has been shown to provide angiogenic, antioxidative, and immunoregulatory effects that accelerate bone regeneration in vivo [96]. Improved hydrophilicity resulting from hydrogel coatings further enhances cell adhesion, promotes osteogenic and angiogenic activity, and reduces inflammation, thereby supporting vessel–bone interactions and new tissue formation [96].

**Figure 4:**
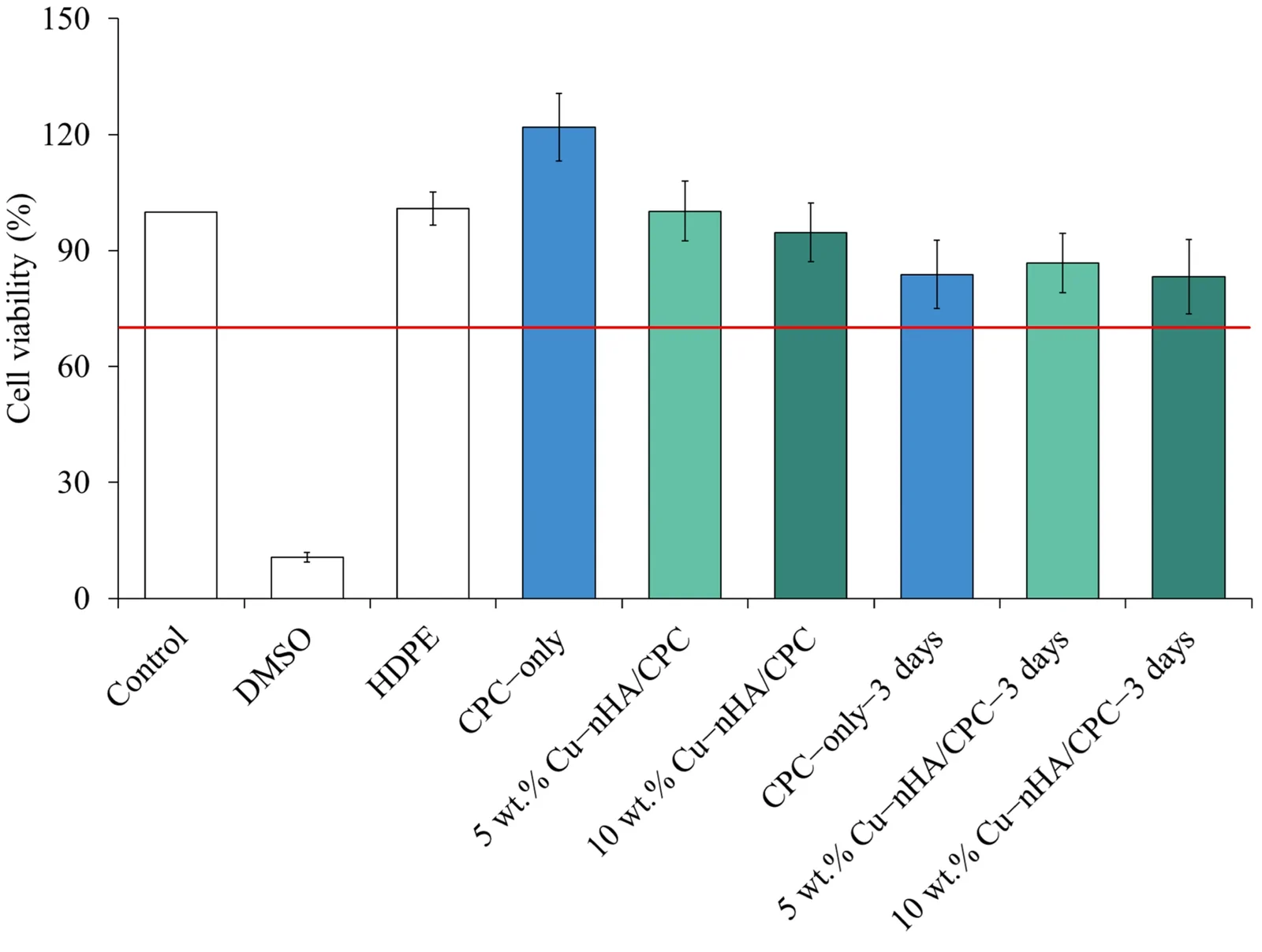
Cytotoxicity of 5 wt.% Cu–nano-HAp/CPC, 10 wt.% Cu–nano-HAp/CPC, and CPC-only extracts was compared to evaluate their effect on L929 cells after 1 day and 3 days of culture. Quantitative measurements were taken. The red line indicates that if the cell viability is less than 70% compared to the control, the substance extract is toxic to the cells. Reproduced with permission from [97].

Copper-doped nano-HAp composites incorporated into calcium phosphate bone cement demonstrated enhanced proliferation and mineralization of osteoblastic cells (Figure 4) [97].

**Figure 5:**
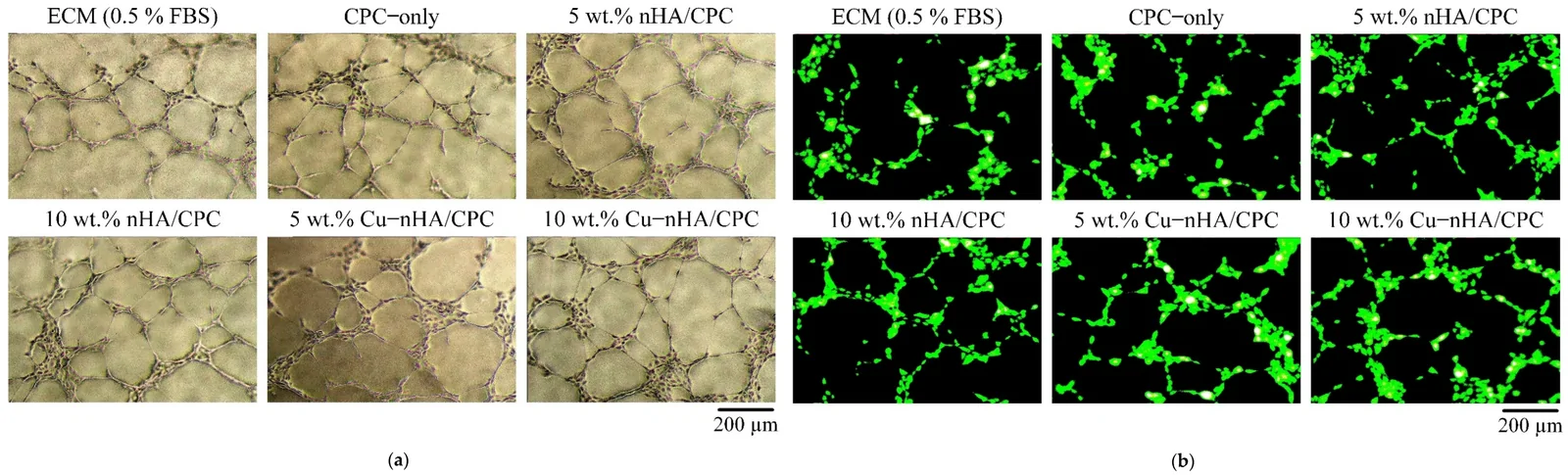
After a 6 h culture of HUVECs with nano-HAp/CPC composite extracts, angiogenesis was assessed through optical (**a**) and fluorescence staining (**b**), with or without Cu^2+^ doping. Reproduced with permission from [97].

These composites also promoted the migration and angiogenic activity of vascular endothelial cells (HUVECs, Figure 5), indicating that copper-containing systems can effectively support vascularization and bone regeneration [97].

Similarly, Zn ion doping has been reported to enhance osteoblast proliferation and angiogenesis, although excessive concentrations may negatively influence thermal stability [91,93].

Reviews on europium-containing biomaterials describe their osteogenic, angiogenic, neuritogenic, and antibacterial properties, while Eu(OH)_3_ nanoparticles in particular have demonstrated marked angiogenic activity relevant to vessel–bone interactions [98]. Composite hydrogels based on gallic acid-grafted chitosan and polydopamine-modified HAp have also shown improved cellular affinity and enhanced bone marrow stem cell differentiation, with newly formed bone characterized by dense trabecular structures and improved vascularization [89]. These findings emphasize the importance of incorporating pro-angiogenic ions and bioactive factors into HAp scaffolds to support functional bone healing and tissue integration.

Ion-doped HAp systems containing Sr^2+^, Zn^2+^, and Cu^2+^ further expand the therapeutic potential of HAp for angiogenesis modulation, particularly in drug delivery systems and implant surface coatings [91]. In animal studies, dual cross-linked gradient COL1/HAp scaffolds loaded with stem cells promoted bone formation, fibrocartilage development, and tissue vascularization, contributing to improved interface healing and vessel–bone crosstalk [84]. Sodium-substituted HAp composites additionally exhibited substantial apatite formation and increased ALP activity, supporting their osteogenic and angiogenic potential [90].

Self-healing HAp-based hydrogels containing magnetic nanoparticles together with Zn-, Mn-, and Ag-doped bioactive glass nanoparticles displayed high swelling capacity, good toughness, and antimicrobial activity [99]. Bioactivity studies further confirmed their bone-bonding ability and capacity to enhance cell proliferation, supporting their use in advanced bone regeneration strategies [99]. Reviews on ion-doped HAp also indicate that strontium, cobalt, and nickel incorporation can improve tissue engineering performance and vascularization [95].

Composite wound dressings incorporating Ag-doped HAp and tetracycline delivery systems have demonstrated accelerated healing, high biocompatibility, and reduced microbial growth, while in vivo studies confirmed both angiogenic and antimicrobial activity [100]. In addition, biphasic Na-HAp/diopside composites showed favorable bioactivity, biocompatibility, and mechanical stability, supporting angiogenesis and bone regeneration in hard tissue engineering applications [90].

#### 3.3.4. Antibacterial Properties

Ion-doped HAp, particularly when modified with Ag^+^, Zn^2+^, Cu^2+^, or Eu^3+^, has demonstrated pronounced antibacterial activity, which is highly relevant for reducing implant-associated infections and supporting bone regeneration processes [91]. Ag-doped HAp has been reported to suppress bacterial growth by more than 95% at a concentration of 1 wt.%; however, higher Ag contents may negatively affect cell viability, emphasizing the need for careful dose optimization [91]. Zn-doped HAp whiskers incorporated into polycaprolactone-based composites also exhibit enhanced antimicrobial activity while maintaining favorable cytocompatibility toward fibroblasts and osteoblasts, supporting their suitability for biomedical applications [101].

**Figure 6:**
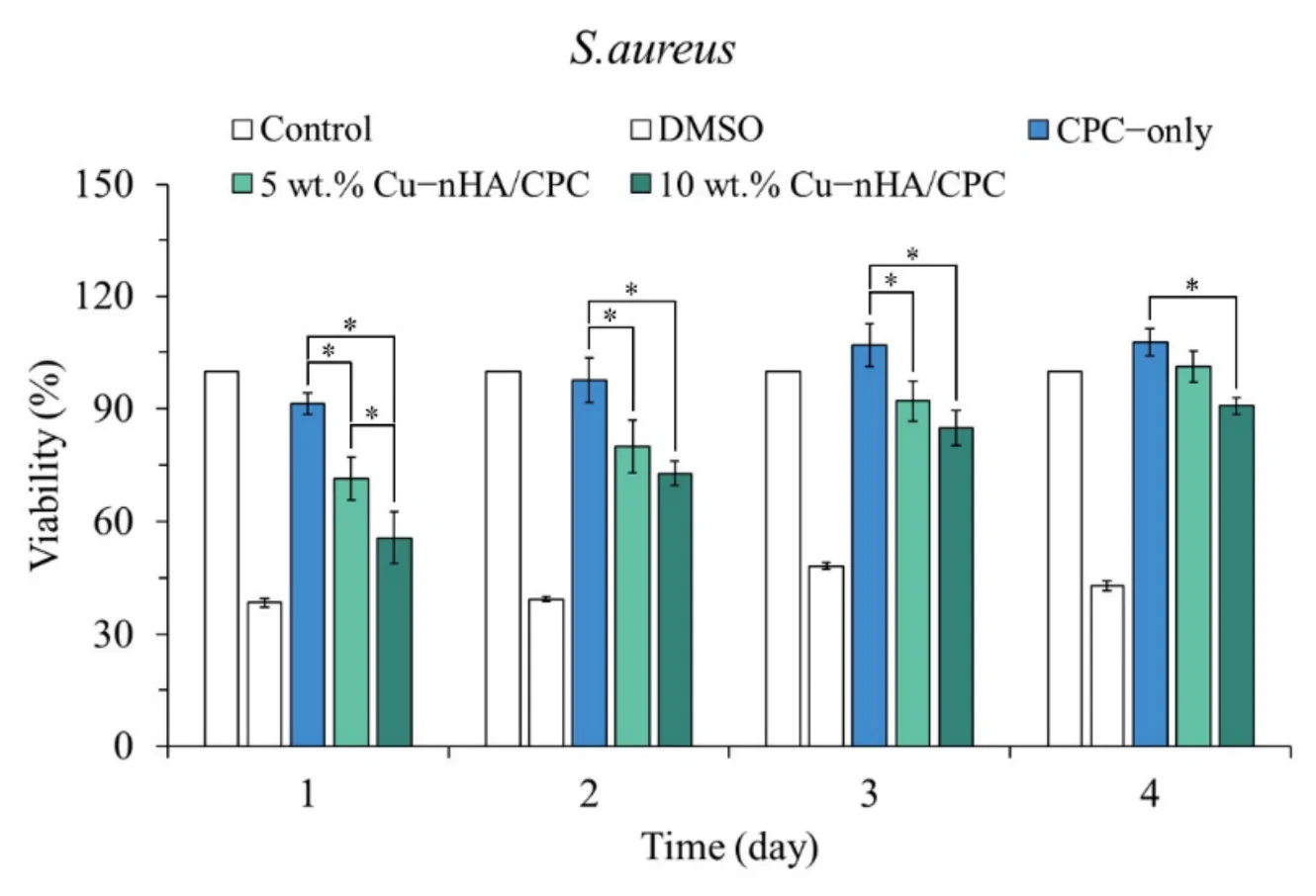
Antibacterial activity of CPC only, 5 wt.% Cu–nano-HAp/CPC, and 10 wt.% Cu–nano-HAp against *S. aureus* cultured for 4 days. * Indicates that the groups were significantly different (*p* < 0.05) based on one-way ANOVA (n = 3). Reproduced with permission from [97].

Copper-doped nano-HAp composites used in bone cement formulations were demonstrated to exhibit short-term antibacterial activity against *S. aureus* (Figure 6) while simultaneously promoting osteogenesis and angiogenesis, highlighting their multifunctional role in bone healing [97].

Similarly, Ag nanoparticle-decorated HAp synthesized through green biosynthetic approaches using plant extracts demonstrates effective antibacterial effects against *E. coli* and *S. aureus*, together with enhanced bioactivity and improved apatite-forming ability [101]. Composite wound dressings containing Ag-doped HAp combined with tetracycline delivery systems have shown high biocompatibility, complete degradation, and substantial inhibition of microbial growth, indicating promising potential for wound-healing applications [100].

Zn-doped HAp nanorods developed for alveolar bone regeneration were shown to exhibit both antibacterial and osteogenic functions by promoting osteogenic differentiation while reducing infection risk, although additional in vivo studies are still required [93].

Eu-containing biomaterials have also attracted attention because of their antibacterial and antitumor properties, with Eu(OH)_3_ nanoparticles demonstrating activity against several pathogens while supporting bone repair processes [98].

More broadly, the antibacterial behavior and drug-loading performance of ion-doped HAp strongly depend on the incorporated dopant, supporting the use of these materials in advanced wound-healing systems and implant coatings [95].

**Figure 7:**
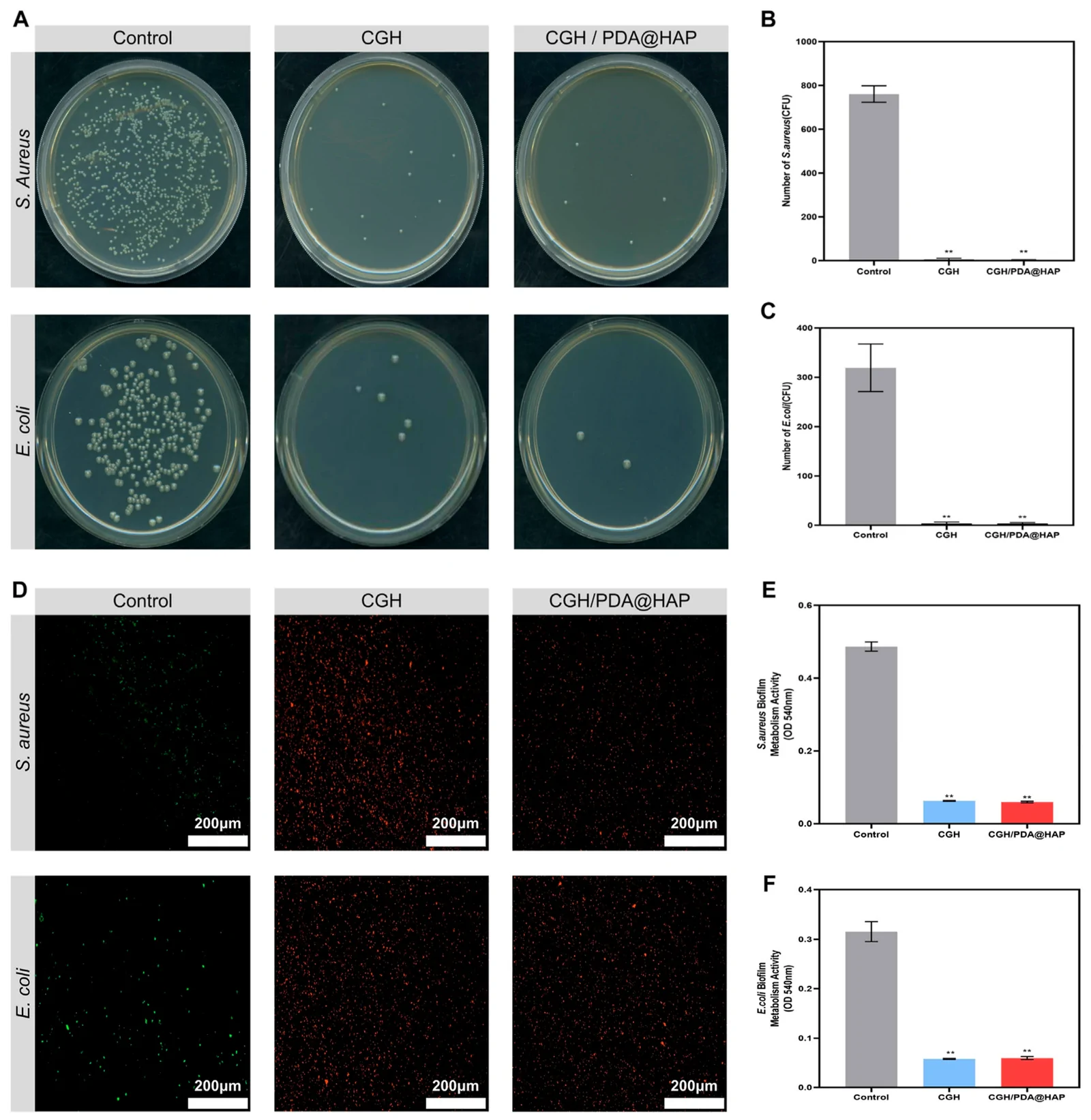
Antibacterial properties of the CGH/PDA@HAp and CGH hydrogels against *S. aureus* and *E. coli*. (**A**) Typical images of *S. aureus* and *E. coli* bacterial colonies after culture with CGH/PDA@HAp and CGH hydrogels. (**B**,**C**) Quantitative analysis of (**A**). (**D**) Live/dead bacteria staining to evaluate the capability of CGH/PDA@HAp and CGH hydrogels to prevent adhesion of *S. aureus* and *E. coli*. (**E**,**F**) MTT assay to assess the effects of CGH/PDA@HAp and CGH hydrogels on the metabolic activity of *S. aureus* and *E. coli* biofilms ( ** *p* < 0.01). Reproduced with permission from [89].

Composite hydrogels based on gallic acid-grafted chitosan and polydopamine-modified HAp effectively inhibit bacterial proliferation (Figure 7) while promoting new bone formation through the combined action of antimicrobial and osteogenic mechanisms [89].

Chitosan-HAp composites are also recognized for their bioadhesive and antibacterial characteristics, with interfacial interactions such as hydrogen bonding, electrostatic attraction, and Ca^2+^ coordination contributing to improved stability and surface bioactivity [82]. Additional modifications, including crosslinking, ion doping, and polymer blending, further allow modulation of degradation behavior, antibacterial activity, and drug-release profiles, thereby supporting the clinical translation of these systems for bone repair applications [82].

Biphasic Na-HAp/diopside composites have demonstrated considerable antimicrobial activity against *P. aeruginosa* and *A. Niger*, together with favorable hemocompatibility, supporting their potential use in hard tissue engineering [90]. Composite hydrogels containing magnetic and bioactive nanoparticles also exhibit antibacterial effects against both Gram-positive and Gram-negative bacteria, suggesting their applicability in advanced bone-related therapies [99]. In addition, HAp-TiC-Ag nanocomposites display strong antibacterial performance against tested bacterial strains while maintaining mechanical properties suitable for bone regeneration and potential anticancer applications [94].

Taken together, these advances highlight HAp as a multifunctional biomaterial capable of meeting the structural, biological, and clinical demands of bone repair and regeneration, while supporting the development of new therapeutic strategies for orthopedic and dental applications.

## 4. Hydrogels for Healing Applications

Hydrogels are crosslinked polymer networks capable of imbibing large quantities of water while maintaining a three-dimensional structure, which gives them a soft, tissue-like character highly suitable for healing and regenerative applications [102,103,104]. Their high-water content, porosity and hydrophilic nature enable efficient transport of nutrients, metabolites and signaling molecules, closely mimicking the native extracellular matrix (ECM) and supporting cell adhesion, proliferation and differentiation in damaged tissues, including cartilage [102,103,105,106]. For healing purposes, hydrogels can function as bioactive dressings, injectable scaffolds or drug-delivery depots, providing a moist environment, mechanical protection and controlled presentation of therapeutic cues that collectively modulate inflammation, angiogenesis and matrix remodeling [103,107,108].

### 4.1. Hydrogel Fundamentals

From a fundamental standpoint, hydrogels are three-dimensional networks formed by physical or chemical crosslinking of hydrophilic polymers, which may be natural, synthetic or hybrid in origin [102,109]. Classification schemes reflect this diversity and typically consider polymer source (natural, synthetic, hybrid, and semi-synthetic), crosslinking mechanism (physical versus chemical), ionic character, architecture (homopolymeric, copolymeric, interpenetrating networks) and responsiveness to environmental stimuli [104,110]. For healing applications, the most relevant distinction is by composition, because polymer identity dictates degradability, bioactivity, mechanical performance and immunological profile [106].

Natural hydrogels are derived from biopolymers such as alginate, chitosan, gelatin, collagen and hyaluronic acid, all of which have been widely explored in wound healing and tissue engineering [105,111].

Alginate, extracted from brown algae, forms ionic gels in the presence of divalent cations; it is biocompatible, gently gels in situ and offers excellent water retention, making it suitable for exudate management, but it lacks intrinsic cell adhesion and typically requires modification or blending to support robust tissue regeneration [112].

Chitosan, obtained by deacetylation of chitin, provides antimicrobial activity, hemostatic effects and cationic functionality that favors interaction with negatively charged cell membranes and ECM components; its solubility and mechanical properties are tuned through degree of deacetylation and crosslinking strategy, enabling applications ranging from wound dressings to injectable regenerative matrices [113].

Gelatin, a denatured derivative of collagen, retains cell-binding motifs (such as RGD sequences) and degrades enzymatically, supporting cell infiltration and matrix deposition; its thermoreversible gelation is often stabilized via secondary crosslinking (e.g., methacrylation) to achieve mechanical integrity under physiological conditions [114].

Collagen itself, the dominant structural protein of native ECM, provides excellent bioactivity and guides cell organization, but its hydrogels are typically mechanically weak and subject to batch variability, requiring reinforcement or hybridization for load-bearing indications such as cartilage repair [115].

Hyaluronic acid, a glycosaminoglycan abundant in cartilage and synovial fluid, contributes to water retention, viscoelasticity and cell signaling. Hyaluronic acid-based hydrogels promote cell migration and matrix remodeling but, like collagen, usually need chemical modification to adjust degradation and strength for regenerative applications [116].

Synthetic hydrogels, exemplified by polyethylene glycol (PEG), polyvinyl alcohol (PVA) and PLGA-based systems, are based on human-made polymers obtained by controlled polymerization of defined monomers [117,118,119]. PEG hydrogels are widely used because PEG is hydrophilic, largely bioinert and can be easily functionalized with reactive groups for crosslinking and with peptides or growth factors to introduce bioactivity; by varying molecular weight, functionality and crosslink density, PEG networks can be engineered with predictable mechanical properties and degradation behavior [120,121,122]. PVA forms physically or chemically crosslinked hydrogels with good mechanical strength and stability; its networks are typically non-degradable unless copolymerized or blended with degradable components, and repeated freeze–thaw cycles or chemical crosslinkers are used to tune elasticity and toughness for applications like load-bearing scaffolds or wound dressings [123,124].

PLGA-based systems generally employ PLGA as degradable segments or particles within a crosslinked network; the hydrolytic degradation of PLGA provides controlled erosion and release of encapsulated agents, while its hydrophobic character necessitates combination with hydrophilic polymers (such as PEG or natural polysaccharides) to form hydrated gels appropriate for tissue engineering [125,126].

Compared with natural hydrogels, synthetic systems typically offer higher mechanical robustness, reproducibility and precise control over architecture, but they lack intrinsic biological recognition and may require surface modification or incorporation of natural polymers to ensure favorable cell–material interactions [110,118].

Hybrid hydrogels combine natural and synthetic components, or multiple natural polymers, to synergistically integrate bioactivity with mechanical performance and structural stability [107,117,127]. This intermediate design is particularly relevant to cartilage and osteochondral repair because unmodified natural polymers often provide favorable cell interactions but insufficient mechanical stability, whereas fully synthetic matrices offer reproducibility and tunability but may lack intrinsic biological cues.

**Figure 8:**
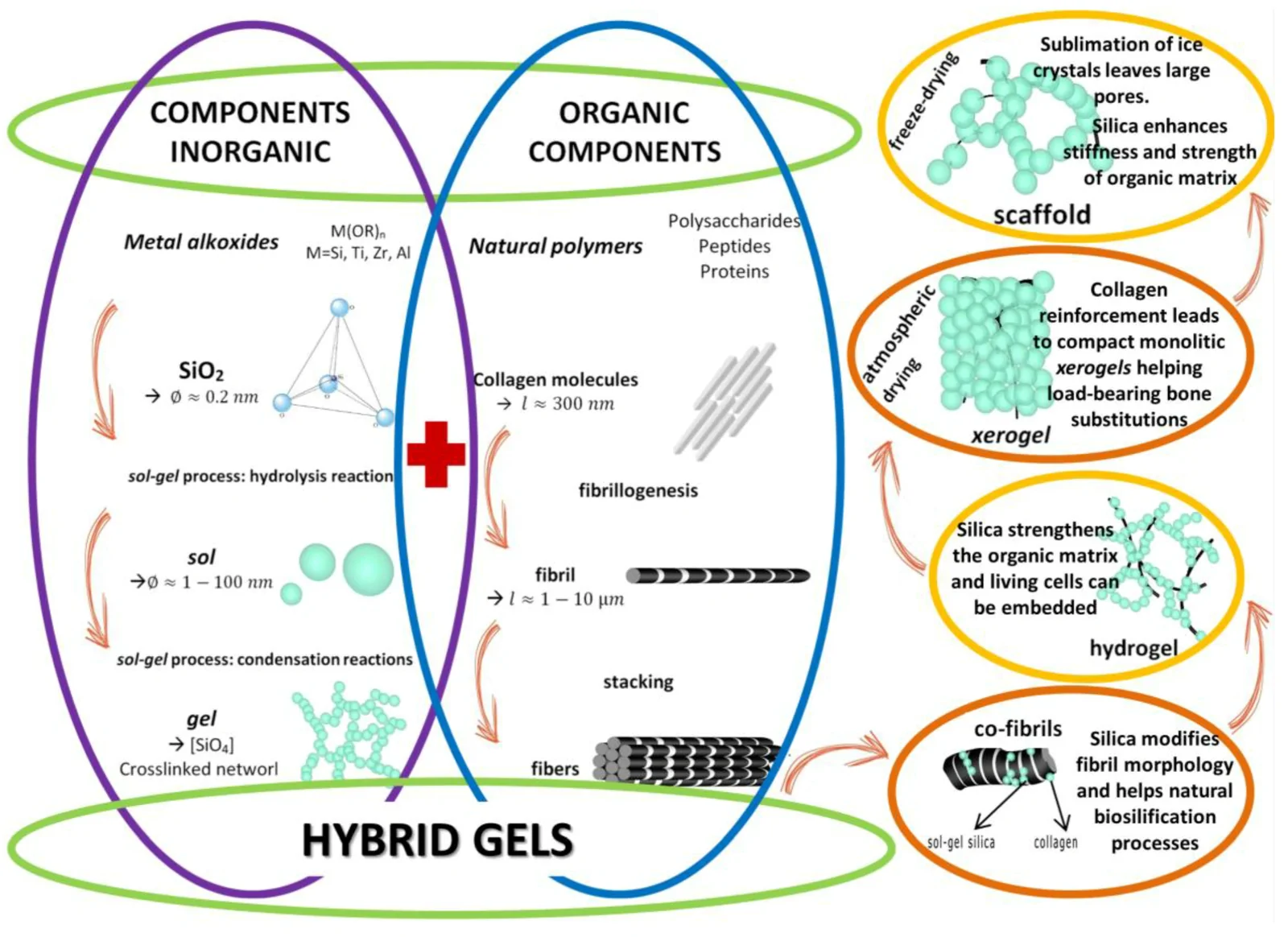
Advantages of hybrid gels: ranging from soft, mineral-rich hydrogels to rigid, compact xerogels. Reproduced with permission from [117].

As summarized in Figure 8, hybrid gel systems bridge the gap between soft mineralized hydrogels—ideal for cell encapsulation and conformable wound coverage—and hard compact xerogels, which provide enhanced mechanical strength and storage stability for chronic wound applications.

At a compositional level, hybrids can be simple blends, copolymeric networks or interpenetrating systems that separately crosslink two or more polymer phases [127]. For regenerative healing, hybrid designs such as gelatin–alginate, chitosan–hyaluronic acid and collagen–chondroitin sulfate seek to recapitulate key ECM features while exploiting complementary strengths: for example, gelatin–alginate matrices marry the cell adhesion of gelatin with the gentle ionic gelation and exudate-handling of alginate [128], whereas chitosan–hyaluronic acid networks combine antimicrobial and hemostatic properties with high water retention and native glycosaminoglycan signaling [129]. In cartilage-oriented scaffolds, hybrid hydrogels incorporating collagen or GelMA with synthetic polymers like PEG or with cartilage-specific glycosaminoglycans (e.g., chondroitin sulfate) can reproduce the viscoelasticity of native cartilage while supporting chondrocyte viability and ECM production [130,131]. Hybridization thus provides a versatile route to engineer matrices whose degradation kinetics, mechanical properties and biological performance are finely matched to the demands of tissue regeneration [132].

The classification of hydrogels as either natural or synthetic is useful at a basic level, but it does not adequately represent the increasing importance of semi-synthetic and hybrid formulations in cartilage tissue engineering. Semi-synthetic hydrogels are generally derived from naturally occurring polymers that have been chemically functionalized—for example, gelatin methacryloyl (GelMA), methacrylated hyaluronic acid (HAMA or MeHA), methacrylated chondroitin sulfate, or oxidized alginate—so that their native biological recognition motifs are retained while their gelation, mechanical strength, degradation, and processing characteristics can be more precisely controlled [133,134].

GelMA is obtained by introducing methacryloyl groups into gelatin, thereby combining the cell-interactive features inherited from collagen with the ability to form covalently crosslinked networks by light-initiated polymerization. Its stiffness, porosity, swelling, and degradation can be adjusted through the GelMA concentration, degree of functionalization, photoinitiator content, and irradiation conditions. These characteristics have made GelMA one of the most widely investigated semi-synthetic matrices for cartilage engineering and 3D bioprinting. Nevertheless, GelMA hydrogels alone are often softer and less resistant to prolonged loading than native articular cartilage; they are therefore frequently reinforced with other polymers, nanomaterials, or mineral phases, including HAp in osteochondral constructs [133,135].

HAMA or MeHA, is produced by functionalizing hyaluronic acid with photocrosslinkable methacrylate or methacryloyl groups. This modification is especially relevant to cartilage regeneration because hyaluronic acid is an endogenous component of cartilage extracellular matrix and can interact with cell-surface receptors while supporting chondrocyte activity and mesenchymal-stem-cell chondrogenesis. Methacrylation provides greater control over gel formation, mechanical behaviour, swelling, and enzymatic degradation than is generally possible with unmodified hyaluronic acid. At the same time, these properties remain sensitive to the polymer molecular weight, degree of substitution, concentration, and crosslinking conditions; highly crosslinked networks may improve dimensional stability but can restrict cell spreading, nutrient transport, or matrix deposition [134,136].

Recent evaluations of HAp-based systems further show that many of the more effective experimental constructs for full-thickness cartilage repair are composite or chemically modified hydrogels, including methacrylated HAp combined with GelMA, chitosan, chondroitin sulfate, or synthetic multifunctional crosslinkers [134]. Accordingly, a more representative classification should distinguish among unmodified natural, chemically modified or semi-synthetic, fully synthetic, and hybrid/composite hydrogels, while recognizing that the boundaries between these groups may overlap depending on composition and network design.

Advanced HAp–hydrogel composites may also be grouped according to their principal therapeutic role. This functional distinction is important because composites intended for cartilage repair, controlled drug delivery, or osteochondral regeneration must satisfy different design and processing criteria. Accordingly, their composition, internal architecture, mechanical behavior, degradation profile, bioactive-agent release characteristics, and fabrication method should be tailored to the requirements of the intended application [137,138].

In mineral-reinforced scaffolds, calcium phosphate phases such as HAp are incorporated into the polymeric matrix to introduce osteoconductive character and, in some formulations, to improve mechanical performance. The resulting properties depend on the mineral content and its spatial distribution, as well as on the composition and crosslinking of the surrounding matrix. Particle size, particle–matrix bonding, dispersion homogeneity, and crosslink density were identified as a common set of dominant design variables across HAp–hydrogel-based systems [44,139].

A spatially organized architecture is particularly relevant to osteochondral repair because articular cartilage, calcified cartilage, and subchondral bone differ in their composition, cellular populations, porosity, and mechanical behavior. To reproduce these depth-dependent features, scaffolds have been developed with biphasic, multiphasic, or continuous-gradient structures using methods such as casting, freeze-drying, phase separation, photopolymerization, and additive manufacturing. In mineral-gradient constructs, the calcium phosphate content may be increased toward the bone-facing region, as illustrated by collagen–HAp scaffolds in which HAp concentration and stiffness rise progressively from the cartilage-like zone to the bone-like zone [44,139,140].

For example, a collagen–HAp scaffold with continuous gradients in mineral content and stiffness supported region-dependent proliferation of mesenchymal stem cells under chondrogenic and osteogenic culture conditions. This suggests that its compositionally distinct regions may provide environments suited to cartilage- and bone-related cellular activity. In a separate approach, a hierarchical HAMA-based construct combined a β-TCP- and strontium-folate-containing porous support with a zinc-folate-loaded hydrogel phase and an intermediate transition zone. This spatial organization promoted preferential colonization by chondrocytes and osteoblasts in vitro and was associated, in a rabbit osteochondral-defect model, with cartilage-like tissue formation at the articular surface and new bone formation in the subchondral region [44,139].

A further group comprises hydrogel-based systems used to deliver cells or bioactive agents, including drugs, growth factors, extracellular vesicles, and, in selected formulations, therapeutic ions. Their design must therefore account not only for mechanical performance but also for payload retention, cell viability, matrix degradation, and the duration and localization of release [141,142]. Stimulus-responsive formulations extend this concept by adjusting drug release in response to changes in temperature, reactive oxygen species, or other features of the local environment [141]. Multifunctional hydrogels have also been investigated for localized antimicrobial delivery, including systems carrying antibiotics, nanoparticles, or bacteriophages to sites of bone infection [142]. Overall, the mineral content, network chemistry, degradation profile, scaffold architecture, and processing route should be selected in relation to the intended therapeutic role rather than treated as components of a universally applicable formulation [141,142].

### 4.2. Crosslinking Mechanisms

The crosslinking mechanism defines the internal architecture of hydrogels and exerts a primary influence on their mechanical behavior, swelling, degradation and biofunctionality, all of which are critical for healing applications [109,143,144]. Broadly, crosslinking strategies can be grouped into physical, chemical, enzymatic, photo-initiated and ionic or dual network approaches, with many clinically oriented systems employing combinations to balance injectability, structural stability and biological compatibility [145].

To achieve the desired mechanical stability and biological functionality for tissue healing, various crosslinking strategies—ranging from reversible physical interactions to permanent covalent bonds—can be employed.

Physical crosslinking relies on reversible, non-covalent interactions such as hydrogen bonding, hydrophobic associations, chain entanglements, crystallization and electrostatic interactions to form the network [109]. Examples include PVA gels obtained by repeated freeze–thaw cycles that induce microcrystalline domains, thermoresponsive polymers that gel upon temperature change, and polysaccharide or protein hydrogels stabilized by hydrogen bonds or hydrophobic interactions [104,146]. Because these interactions are dynamic, physically crosslinked hydrogels often exhibit shear-thinning behavior and the capacity to self-assemble or disassemble in response to environmental stimuli, properties that favor minimally invasive injection and in situ formation without the need for exogenous crosslinkers [147]. However, their mechanical strength and long-term stability are typically lower than covalently crosslinked systems, necessitating careful design when used in mechanically demanding sites such as articulating cartilage [102].

Chemical crosslinking creates covalent bonds between polymer chains, yielding hydrogels with higher mechanical strength, improved dimensional stability and more predictable degradation [144]. Covalent networks can be formed by small-molecule crosslinkers (e.g., aldehydes, carbodiimides, multifunctional acrylates), by click-chemistry reactions (such as thiol-ene or azide–alkyne cycloadditions), or by condensation reactions between reactive functional groups introduced onto the polymer backbone [145]. Polysaccharides and proteins are commonly crosslinked with agents like genipin, carbodiimides or multifunctional PEG derivatives to form robust networks, while synthetic polymers such as PEG diacrylate are crosslinked via radical polymerization [109]. For healing applications, chemical crosslinking allows the design of hydrogels that withstand physiological loads, provide sustained structural support and display controlled, often hydrolysis- or enzyme-mediated, degradation profiles that can be matched to the time course of tissue regeneration [109,145]. Safety considerations drive the shift toward bio-orthogonal and low-toxicity chemistries, particularly when scaffolds are formed in situ [102].

Enzymatic crosslinking uses enzymes such as horseradish peroxidase, transglutaminase, tyrosinase or laccase to catalyze bond formation between suitably functionalized polymers, enabling gelation under mild, physiological conditions [148]. For example, phenol- or catechol-modified polymers can be crosslinked by horseradish peroxidase in the presence of hydrogen peroxide, while transglutaminase catalyzes bonds between glutamine and lysine residues in protein- or peptide-containing matrices [149]. These reactions are highly specific and can be temporally controlled by adjusting enzyme and substrate concentrations, which is advantageous for in situ formation around delicate tissues, encapsulated cells or bioactive factors. In healing contexts, enzymatically crosslinked hydrogels have been used as bio-adhesives, injectable scaffolds and drug carriers that rapidly conform to irregular defects while exhibiting good biocompatibility and tailored degradation [150].

Photo-crosslinking, typically based on radical polymerization of (meth)acrylated or other photo-reactive groups, offers spatiotemporal control over network formation and is widely used for hydrogels in tissue engineering, including cartilage [151]. Upon exposure to UV or visible light in the presence of a photoinitiator, reactive moieties on the polymer chains form covalent crosslinks, allowing rapid gelation and patterning with high precision [152,153]. Photosensitive derivatives of alginate, chitosan, gelatin and PEG (e.g., GelMA, PEG-DA, methacrylated alginate) have been developed to enable in situ cured scaffolds, microstructured constructs and 3D-printed architectures that mimic complex tissue geometries [154]. For healing applications, the ability to modulate light intensity, exposure time and initiator type permits fine tuning of crosslink density and, consequently, mechanical properties, swelling and degradation, though care must be taken to minimize phototoxicity and ensure adequate light penetration in vivo [152].

Ionic crosslinking and dual-network strategies further expand the design space for hydrogels used in healing. Ionic networks are formed by electrostatic interactions between charged polymers and counterions, as seen in alginate gels crosslinked with calcium or in polyelectrolyte complexes between oppositely charged polysaccharides [155]. These interactions are reversible and responsive to ionic strength and pH, affording gels that can be injected as solutions and solidify upon exposure to physiological ions or that gradually relax and remodel in vivo [156]. Dual-network hydrogels combine two interpenetrating networks, often pairing a brittle but densely crosslinked primary network with a softer, ductile secondary network to achieve high toughness and resilience [155,157,158]. In healing applications, including cartilage and bone repair, dual and interpenetrating networks can reconcile the conflicting demands of high mechanical strength, fatigue resistance, injectability and cell friendliness, and can be further functionalized with self-healing motifs through dynamic covalent or supramolecular interactions [156,159].

Crosslinking agents play a critical role in determining hydrogel performance. Depending on their chemical nature, they can strongly influence wettability, mechanical strength, degradation rate, swelling behavior, and cytocompatibility [160]. A higher crosslink density usually improves structural stability and reduces solubility, but it may also decrease pore size, limit diffusion, and restrict cell infiltration. In contrast, milder and more biocompatible crosslinking strategies may better preserve cell viability while still providing sufficient network integrity for biomedical use [161]. Therefore, the crosslinking approach should always be selected according to the intended application and the desired balance between mechanical support and biological compatibility [162].

### 4.3. Functional Properties for Healing

The success of hydrogels in healing applications depends on a constellation of functional properties—swelling behavior, mechanical strength, degradation kinetics, injectability, self-healing capacity and biocompatibility—that must be tuned in an integrated manner rather than in isolation [108].

The mechanical performance of hydrogel-based composites depends strongly on the selected fabrication method, polymer composition, and crosslinking density. Chemical crosslinking generally increases stiffness and structural stability, whereas physical gelation may provide greater flexibility but lower mechanical resistance [163]. In contrast, dual-network systems and reinforced composites often exhibit improved toughness and compressive strength, which are particularly important for cartilage and osteochondral applications [164]. Accordingly, the fabrication strategy should be selected based on the mechanical requirements of the target tissue and the intended biological function of the material [165].

To guide the rational design of HAp–hydrogel composites, it is essential to first understand the fundamental physicochemical properties of the hydrogel matrix that directly govern healing outcomes, which are summarized in Table 2.

Swelling behavior arises from the hydrophilic nature of the polymer network and the balance between osmotic driving forces and elastic retraction of the crosslinked chains [166]. For wound dressings and regenerative scaffolds, sufficient swelling is essential to maintain hydration, absorb exudate and allow diffusion of oxygen, nutrients and signaling molecules, while excessive swelling can compromise mechanical stability, cause maceration of surrounding tissues or alter local mechanical cues critical for processes like chondrogenesis [183]. Crosslink density, polymer charge and composition govern equilibrium water content and swelling kinetics, and these parameters are carefully adjusted to match the moisture balance and mass-transport needs of the target tissue environment [184].

Mechanical strength and viscoelastic properties must be compatible with both the implantation site and the dynamic mechanical environment associated with healing [185]. Hydrogels for superficial wound care can be relatively soft and flexible while still providing protection, whereas hydrogels intended for cartilage regeneration must exhibit compressive modulus, toughness and fatigue resistance closer to native cartilage to support joint loading without failure [171]. These properties are regulated primarily by the type and density of crosslinks, polymer backbone stiffness, network architecture and the presence of reinforcing phases (e.g., fibers, nanoparticles or a second network) [186,187]. Chemical or dual-network crosslinking strategies often yield the required mechanical robustness, and the incorporation of synthetic polymers or inorganic fillers into natural matrices is a common approach to overcome the inherent weakness of purely natural hydrogels [188,189,190,191]. In healing contexts, mechanical cues transmitted through the hydrogel also influence cell fate decisions, including differentiation of progenitor cells toward chondrogenic or osteogenic lineages, highlighting the need for finely tuned stiffness and viscoelasticity [192,193]. Recent work using stiffness-tuned hyaluronic acid–gelatin hydrogels further demonstrated that MSC osteogenic differentiation depends on an optimal mechanical range rather than simply increasing matrix stiffness. Hydrogels with Young’s moduli of 3.3, 6.0, and 10.1 kPa produced different cellular responses, with the intermediate-stiffness hydrogel (6.0 kPa) inducing the highest expression of osteogenic markers, whereas the stiffest matrix primarily enhanced YAP nuclear localization. These findings also enabled the formation of transferable MSC-derived osteogenic cell sheets, supporting the use of stiffness-controlled hydrogels as cell-instructive regenerative platforms [194].

The reported mechanical values should be interpreted in relation to the requirements of the target tissue. For context, the equilibrium compressive modulus of healthy articular cartilage is commonly reported in the range of approximately 0.1–2.0 MPa [195], with aggregate moduli typically around 0.5–0.9 MPa, while the tissue experiences in vivo compressive stresses on the order of 1–2 MPa [196] during routine loading. For cartilage-related applications, an appropriate balance between stiffness, elasticity, and resilience is essential to withstand repetitive loading without structural failure [197]. If the measured compressive modulus or strength is below the range expected for functional cartilage support, the material may still be suitable for early-stage regeneration or non-load-bearing environments, but additional reinforcement would be required for long-term mechanical performance. By contrast, values approaching the desired range indicate a more promising potential for structural repair and osteochondral support [198].

Degradation kinetics must be synchronized with the time course of tissue repair and the intended therapeutic function of the hydrogel, so that material resorption matches tissue remodeling and avoids either premature loss of support or prolonged foreign-body presence [199].

Biodegradable natural polymers such as collagen, gelatin and hyaluronic acid are primarily degraded by specific enzymes (e.g., collagenases, matrix metalloproteinases, hyaluronidases), whereas synthetic polyesters such as PLGA and related materials undergo hydrolytic cleavage of ester bonds; in contrast, neutral synthetic polymers like PEG and PVA are intrinsically non-degradable in vivo unless modified with labile linkages or combined with degradable segments [200].

For healing applications, particularly in regenerative medicine, the hydrogel should maintain structural integrity long enough to provide mechanical support and deliver bioactive molecules or cells, yet gradually degrade to permit cell infiltration, extracellular matrix deposition and eventual restoration of native tissue architecture; inappropriate timing (too fast or too slow) can impair regeneration or provoke chronic inflammation [201].

Crosslink type, crosslink density, polymer composition and the incorporation of labile chemistries (e.g., ester, acetal, thioester, or enzymatically cleavable peptide crosslinks) are key design levers to tailor degradation profiles from days to months and to couple degradation to cell-mediated proteolysis or environmental cues [202,203]. Importantly, degradation products must be non-toxic, non-immunogenic and readily cleared to avoid chronic inflammation or systemic toxicity, which requires attention not only to the base polymer but also to crosslinkers, initiators and by-products of hydrolytic or enzymatic cleavage [204].

Injectability is a particularly valuable property for hydrogels designed for minimally invasive delivery into irregular defects, deep wounds or joint spaces, enabling local treatment while reducing surgical morbidity [205]. Injectable hydrogels are generally formulated as low-viscosity precursors that undergo sol–gel transition in situ via thermal, ionic, enzymatic, pH-triggered, photo-initiated or chemical crosslinking mechanisms [206].

Shear-thinning behavior, common in physically or dynamically crosslinked systems, allows the material to flow under the high shear conditions of injection and then rapidly recover its structure once shear is removed, minimizing tissue damage and ensuring defect conformity [207]. For healing applications, injectability enables targeted, patient-specific filling of complex lesions, co-delivery of cells or drugs and repeat dosing if needed, while facilitating clinical translation in drug delivery, wound repair and tissue regeneration [145].

Self-healing capacity, arising from reversible covalent bonds or supramolecular interactions, further enhances the robustness and longevity of hydrogels in dynamic physiological environments [208]. Self-healing hydrogels exploit mechanisms such as dynamic Schiff base (imine) formation, disulfide exchange, boronate ester bonds, host–guest interactions, hydrogen bonding or ionic interactions to autonomously repair microcracks or macroscopic damage, recovering mechanical integrity and barrier function after deformation or trauma [209].

In wound healing and tissue repair, this capacity helps maintain scaffold continuity, prolongs therapeutic function and allows repeated mechanical loading without premature failure, which is especially advantageous for load-bearing tissues and for wearable or implantable devices that experience cyclic stresses [210,211]. Additionally, many self-healing systems exhibit injectability and stimuli-responsiveness, enabling not only minimally invasive administration but also on-demand modulation of drug release or mechanical properties in response to pH, redox conditions, ROS or other biochemical signals associated with healing [212,213]. Biocompatibility underpins all of these functional attributes and is non-negotiable for clinical translation [102,214].

Natural hydrogels generally display favorable biocompatibility and biodegradability, reflecting their origin in endogenous ECM components or structurally related polysaccharides, but they can exhibit batch-to-batch variability, source-dependent impurities and, in some cases, allergenicity or immunological risks that must be carefully controlled [214,215].

Synthetic hydrogels such as PEG and PVA are typically non-toxic and show low protein adsorption and minimal inflammatory response in their pure form, yet they lack intrinsic cell-recognition motifs and often require functionalization with peptides or ECM-derived components to promote integration and avoid fibrous encapsulation or poor tissue remodeling [216,217].

For healing applications, particularly in chronic wounds and cartilage repair, the optimal hydrogel not only avoids acute and chronic toxicity but also actively supports cell adhesion and proliferation, modulates macrophage-driven inflammation, can incorporate antimicrobial agents when needed, and fosters orderly progression through the hemostasis–inflammation–proliferation–remodeling phases toward functional tissue restoration [218]. Achieving this balance increasingly involves the rational design of multifunctional hydrogels that integrate tailored swelling, mechanics and degradation with injectability, self-healing, bioactivity and, in some cases, stimuli-responsive behavior to address the complex, evolving microenvironments encountered during tissue regeneration [218,219].

## 5. Design Strategies for HAp–Hydrogel Composites

HAp–hydrogel composites, ceramic–polymer scaffolds, and bioprinted constructs should be distinguished because they refer to different aspects of scaffold design. HAp–hydrogel composites are defined by the integration of a mineral phase within a highly hydrated polymer network, thereby combining the water-rich and compliant characteristics of the hydrogel with the reinforcing effect of HAp [220]. In osteochondral constructs, spatially distributed nano-HAp can additionally provide mineral and osteogenic cues suited to the subchondral region, while generating region-dependent mechanical and biological properties across the scaffold [29]. Ceramic–polymer scaffolds represent a broader materials category and commonly use relatively rigid thermoplastic or structural polymer matrices reinforced with calcium phosphate ceramics [221]. Such systems may offer greater dimensional stability and load-bearing capacity than soft hydrogels, but they generally provide less intrinsic hydration and are less readily suited to homogeneous cell encapsulation. Three-dimensional bioprinting, in contrast, is a fabrication technology rather than a separate biomaterial class. It enables the spatial placement of hydrogels, ceramics, cells, and bioactive agents within multilayered or gradient constructs designed to reproduce the heterogeneous cartilage–bone unit [138,222]. Therefore, the particular promise of HAp–hydrogel composites for osteochondral regeneration lies in combining a cartilage-compatible hydrated phase with a mineral component suited to subchondral-bone repair, while remaining compatible with bioprinting and other spatially controlled manufacturing methods. However, since direct comparative evidence remains limited, the selection of a hydrogel composite, a rigid ceramic–polymer scaffold, or a printed multiphasic construct should ultimately be guided by the mechanical, biological, and anatomical requirements of the defect.

Designing HAp–hydrogel composites for cartilage regeneration requires tight control over mineral introduction, particle size, interfacial chemistry, and the ability of the construct to respond to biochemical and physical cues in the defect microenvironment. In cartilage- and osteochondral-oriented systems based on chitosan, alginate, gelatin, hyaluronic acid, poly(vinyl alcohol), and related polymers, these variables govern dispersion quality, mechanical reinforcement, viscoelastic behavior under cyclic loading, and chondro/osteogenic activity at the cartilage–bone interface [223]. The most consistently supported design principle is that HAp is not only a filler, but also a bioactive phase whose size, shape, and surface chemistry directly affect cell response and network mechanics [224]. In hydrogel composites developed for cartilage-related applications, HAp may perform functions that extend beyond its role in reproducing the mineralized region of the osteochondral interface. Thus, HAp can influence both the mechanical behavior and biological performance of the material. The extent of these effects, however, varies with the hydrogel composition, HAp content, and the tissue region that the construct is intended to reproduce [220,225,226]. Experimental studies have shown that the incorporation of nano-HAp or HAp nanorods can reinforce selected hydrogel formulations, increasing compressive strength and elastic or tensile properties and, in some systems, improving printability and dimensional stability. These effects are dependent on the HAp concentration, as excessive particle loading may cause aggregation and compromise mechanical performance. It should be mentioned that HAp reinforcement is relevant because unmodified hydrogels often do not possess the mechanical properties needed for cartilage-replacement applications. Even though such reinforcement may improve the mechanical suitability of hydrogel scaffolds for cartilage-replacement applications, their resistance to repeated physiological loading and long-term fatigue has not yet been established [220,225,227].

The reported biological effects also vary with the experimental model. Thus, HAp-containing hydrogels demonstrated acceptable cytocompatibility in studies involving L929 fibroblasts, human mesenchymal stem cells, and chondrocytes, with reported evidence of cell viability, spreading, and proliferation [220,225,226]. However, the available evidence does not establish a general ability of HAp to induce stable chondrogenic differentiation toward non-mineralized articular cartilage. In alginate/HAp systems, the observed response was associated with calcified cartilage formation, as HAp increased collagen type X expression, alkaline phosphatase activity, and mineral deposition. These findings are particularly relevant to regeneration of the calcified cartilage zone and the osteochondral interface. Based on the reported results, HAp should be regarded primarily as a concentration-dependent mechanical reinforcing and bioactive component, with its clearest demonstrated tissue-specific role being the formation of calcified cartilage at the osteochondral interface [226].

### 5.1. Incorporation Methods

Physical blending is the simplest route, in which preformed HAp particles are mixed into a polymer solution before gelation. This method is widely used for nano-HAp in chitosan, alginate, gelatin, and PVA/PVP hydrogels, where the mineral phase acts as both a mechanical reinforcement and a bioactive cue [228,229]. In gelatin methacrylate and sodium alginate systems, dispersed HAp nanoparticles or microparticles can substantially increase compressive strength and Young’s modulus while preserving water content and cytocompatibility [223]. A well-documented example is the nano-HAp-reinforced chitosan composite hydrogel, which showed tunable mechanical and biological properties for cartilage regeneration [228].

In situ mineralization can improve dispersion and the interface by precipitating calcium phosphate inside a preformed or simultaneously forming hydrogel network. This strategy promotes intimate interpenetration between the organic matrix and apatite phase, often improving mechanical properties and osteogenic signaling [230].

A related and especially relevant approach uses hyaluronic acid as a mineralization template to generate HAp-based hybrid particles that can then be incorporated into self-healing hydrogels [231]. In that study, oxidized hyaluronic acid/HAp hybrid particles significantly increased storage modulus and improved injectability while maintaining cytocompatibility [231].

Surface-functionalized HAp is used to improve compatibility between rigid inorganic crystals and soft hydrophilic polymers. Rod-like and fibrous HAp morphologies can strengthen ionic and hydrogen-bonding interactions with chitosan or PVA/PVP networks, increasing storage modulus while retaining rapid recovery under cyclic loading [228]. These anisotropic particles can also better mimic the architecture of native bone apatite and are associated with improved osteoblast adhesion and alkaline phosphatase activity [228]. For GelMA systems, mineralized HAp nanofibers can improve both mechanics and in vivo bone regeneration, but the loading level must be optimized to avoid brittleness [230].

Core–shell architectures provide another way to engineer the HAp–hydrogel interface. Microfluidic fabrication can generate core–shell microspheres where HAp serves as a bioactive reservoir for sustained release, while the shell controls diffusion and mechanical integrity [230]. Hybrid approaches combining HAp particles with self-healing polymer networks are particularly attractive because they can preserve injectability while improving load-bearing performance [231]. Hierarchical designs, such as thermosensitive hydrogels incorporating nano-HAp and drug-loaded microcapsules, further expand the design space by separating mechanical reinforcement from staged release functions [231].

Hydrogel fabrication methods in the cited literature include physical and chemical gelation strategies, including covalent crosslinking and enzyme-mediated network formation [223], alginate-based hydrogel–mineral composites formed under ambient gelation conditions with HAp incorporation [224], chitosan/nano-HAp composite hydrogels prepared by dissolving chitosan in acetic acid, sonicating HAp nanorods, magnetic stirring, glutaraldehyde crosslinking, molding at low temperature, and ethanol/water washing [228], as well as stiffness-tuned MSC-laden hydrogels used to study mechanoregulation and immunomodulation [231].

The preparation method is an essential parameter because it strongly influences network architecture, pore structure, swelling behavior, mechanical stability, and biological performance [232,233].

By comparing the available incorporation strategies, it is indicated that uniform dispersion of HAp within the hydrogel matrix generally produces more consistent mechanical reinforcement and cellular responses than simple physical mixing, which often results in particle aggregation. Surface-modified HAp further improves particle dispersion and interfacial interactions with polymer networks, thereby enhancing both mechanical integrity and biological performance. Accordingly, recent studies increasingly favor controlled incorporation methods that optimize HAp distribution while preserving hydrogel injectability and structural homogeneity.

### 5.2. Nano-Versus Micro-HAp in Hydrogels

The choice between nano- and micro-scale HAp strongly affects dispersion, network formation, and overall performance. Nano-HAp usually has a higher specific surface area and stronger interfacial interactions with polymers, but it also tends to agglomerate more easily; micro-HAp is easier to process and can accelerate gelation in some systems [228,229]. This distinction matters because cartilage and osteochondral scaffolds often need both high-water content and robust mechanical response.

Particle size also changes gelation behavior and microstructure. In pectin-based systems, coarse HAp particles can accelerate gelling and produce stronger but less homogeneous hydrogels, while nanoscale HAp generally yields more uniform structures with longer and more controllable gelation times [234]. In alginate-based composites, nano-HAp is typically more evenly distributed at moderate loadings, although increasing concentration can still produce localized clusters that correlate with mechanical strengthening [229]. Conversely, micro-HAp can act as a more defined osteoconductive phase, especially in constructs designed to mimic calcified cartilage or trabecular bone [230].

Mechanical reinforcement is also size dependent. Nanorods and nanofibers can bridge pores and form percolated networks that redistribute stress more effectively than equiaxed particles [228]. In chitosan hydrogels, increasing nano-HAp content raises modulus and compressive strength while reducing swelling and degradation, indicating a denser and more stable network [228]. For PVA/PVP hydrogels, nanorod-filled systems can reach compressive strengths in the cartilage-relevant range and show elastic-dominant viscoelastic behavior under cyclic loading [228]. Micro-HAp can still be useful, but its primary advantage is often simpler processing and clearer osteogenic presentation rather than maximal reinforcement [230].

Bioactivity differences between nano- and micro-HAp reflect not only surface area but also morphology and crystallinity. Bone-like nanoscale rods and fibers tend to improve osteoblast adhesion, alkaline phosphatase activity, and osteogenic signaling more strongly than conventional irregular particles [228]. In composite hydrogels, nano-HAp often promotes greater proliferation and differentiation of stromal cells than micro-HAp at equivalent mass fractions while maintaining cytocompatibility [235]. Micro-HAp, by contrast, can provide discrete adhesion sites and a stable mineral phase, but its lower surface area may limit ion-release-driven signaling [230].

Based on the above, the available evidence consistently favors nano-HAp for osteochondral applications. Compared with micro-HAp, nano-HAp exhibits a larger specific surface area, greater protein adsorption capacity, more efficient ion release, and improved interactions with stem cells, resulting in enhanced osteochondral regeneration. However, nano-HAp is also more susceptible to aggregation at high concentrations, emphasizing the importance of appropriate surface functionalization and homogeneous dispersion within the hydrogel matrix. Thus, current research increasingly focuses on optimizing nano-HAp distribution rather than simply reducing particle size.

### 5.3. Smart and Responsive Systems

Beyond static reinforcement, HAp–hydrogel composites can be engineered as smart, stimuli-responsive systems that adapt to the dynamic cartilage defect microenvironment. Stimuli such as pH, enzyme activity, temperature, and magnetic fields can be harnessed to modulate swelling, degradation, mechanical behavior, and the release of growth factors, anti-inflammatory agents, or antibiotics, thereby improving spatiotemporal control over regeneration [236]. This is especially relevant in osteoarthritic lesions, where inflammation, acidic conditions, and enzymatic remodeling coexist.

pH-responsive systems exploit ionizable groups in the polymer network and the pH-dependent chemistry of calcium phosphates. In mineralized hydrogel systems, HAp can also help buffer local acidity through dissolution–reprecipitation processes, which may be beneficial in inflamed joints [237]. More broadly, pH-sensitive mineralized hydrogels are being developed to stabilize the microenvironment and improve tissue regeneration in inflammatory settings [238].

Enzyme-responsive systems introduce remodeling capacity into the scaffold using polymers such as chitosan, gelatin, and hyaluronic acid, which are susceptible to enzymatic cleavage. In such systems, the scaffold gradually degrades in response to matrix remodeling, while HAp maintains osteoconductivity during turnover. Enzymatically degradable chitosan/HAp composites are therefore attractive for bone regeneration and may also be adapted to osteochondral repair where controlled resorption is required [239].

Munarin et al. made a comparison between nano- and micro-HAp that shows clear differences in particle size, dispersion, reinforcement behavior, and biological suitability: nano-HAp has an average grain size of about 25 nm and appears as randomly shaped flocs formed by weak interactions among nanometric particles, whereas micro-HAp consists of large spherical granules ranging from 2 to 40 μm that are made of strongly bonded small grains; in hydrogels, nano-HAp tends to form aggregates and therefore needs carefully controlled gelling kinetics of about 5–8 min to achieve a homogeneous distribution, while micro-HAp disperses well in pectin matrices but shows the longest crosslinking time, exceeding 60 min at pH 3.7. In terms of reinforcement, nano-HAp provides intermediate rheological properties with better temperature stability, while micro-HAp, despite reports of improved mechanical properties in other studies, showed a lower storage modulus than the smaller-particle HAp in this work. Overall, the work suggests that nano-HAp offers the best compromise for tissue engineering because it combines uniform dispersion with suitable gelling times for cell or drug immobilization [240].

Table 3 summarizes incorporation route, mineral scale, main advantages, and key limitations.

Among the available fabrication methods, 3D printing offers major advantages in geometric precision, shape control, and patient-specific scaffold design [244]. It allows the production of complex architectures with controlled pore distribution and spatially resolved composition, which is especially useful for osteochondral constructs [245]. However, 3D printing also requires careful optimization of bioink viscosity, crosslinking kinetics, and post-printing stability [246]. Compared with freeze-drying or conventional casting methods, it provides greater structural reproducibility and more advanced design flexibility, but its suitability still depends on the mechanical requirements, biological performance, and clinical application of the final construct [138].

To better compare the principal fabrication routes used for HAp–hydrogel composites, Table 4 summarizes the main advantages, limitations, mechanical characteristics, and application domains of 3D printing, freeze-drying, crosslinking, and injectable hydrogel systems.

The intended application of a given composite should always be considered together with its fabrication strategy [267]. Injectable systems are particularly suitable for minimally invasive delivery and defect filling, whereas porous scaffolds produced by freeze-drying or 3D printing are better adapted to load-bearing tissue engineering applications. In cartilage and osteochondral repair, the choice of fabrication method must balance injectability, structural integrity, and biological functionality [134]. Therefore, each material system should be interpreted not only in terms of its composition, but also in relation to the clinical context for which it was designed [268]. Taken together, the fabrication method, crosslinking strategy, mechanical profile, and biological context must be considered jointly when evaluating the suitability of HA–hydrogel composites for cartilage and osteochondral regeneration [267].

## 6. Mechanisms of Healing Enhancement

Cartilage regeneration remains a major clinical challenge due to the limited intrinsic repair capacity of cartilage, which arises from its avascular nature, low cell density, and limited progenitor cell recruitment. As a result, untreated defects often progress toward degeneration and OA. It is important to mention that articular cartilage lacks a vascular supply and has a poor capacity for spontaneous repair after injury. This creates a need for scaffolds that can support cell survival and tissue formation while maintaining adequate mass transfer. Hydrogels are attractive for this purpose because their hydrated polymer networks can provide a cytocompatible environment and permit mass transport. Nevertheless, their pore architecture and mechanical properties often require optimization. A further challenge is reproducing the depth-dependent and anisotropic organization of the osteochondral unit. Native osteochondral tissue exhibits gradients in cell type, matrix composition, architecture, and mechanical properties from the articular-cartilage surface through calcified cartilage to subchondral bone. The calcified-cartilage region is particularly important because it provides a transition through which compressive, tensile, and shear forces are transferred from viscoelastic cartilage to the stiffer underlying bone. HAp–hydrogel composites can be organized as multilayered or compositionally graded constructs to approximate these regional differences. For example, trilayer nHAMA/GelMA scaffolds containing cartilage, calcified-cartilage, and subchondral-bone compartments have shown gradient pore dimensions, improved mechanical performance, region-associated chondrogenic and osteogenic activity, and enhanced osteochondral repair in vivo. Such findings support the use of spatially differentiated composite scaffolds to address both the biological heterogeneity of the osteochondral unit and the mechanical demands imposed by the joint environment [220,267].

HAp–hydrogel composites offer a multifunctional platform which addresses these limitations through biomimicry, bioactivity, and controlled microenvironment modulation, enabling a shift from passive repair to active regeneration.

### 6.1. ECM Mimicry and Cell Support

Hydrogels are suited for cartilage regeneration due to their close resemblance to the native ECM, both structurally and functionally. Their high-water content (typically 70–99%), combined with viscoelastic properties and interconnected porous architecture, closely mimics the hydrated, proteoglycan-rich environment of articular cartilage [269]. This biomimetic microenvironment is essential for maintaining cell viability, phenotype stability, and metabolic activity, particularly for chondrocytes, which are highly sensitive to osmotic pressure and mechanical cues [142].

The biological response was evaluated using the cell line or primary cells reported in the original study, including MG-63 osteoblast-like cells [270], MC3T3-E1 pre-osteoblasts [271], L929 fibroblasts [272], HUVECs [273], and mesenchymal stem cells, depending on the specific application [274]. Identifying the exact cellular model is important because proliferation, differentiation, adhesion, and cytocompatibility can vary significantly between cell types [26].

From a mechanobiological perspective, hydrogels can be engineered to replicate the mechanical stiffness and dynamic loading conditions of cartilage, which are known to regulate chondrocyte function and mesenchymal stem cell (MSCs) fate [142]. A properly tuned hydrogel stiffness promotes chondrogenic differentiation of MSCs by activating mechanotransduction pathways (e.g., integrin-mediated signaling), while preventing undesirable hypertrophic or osteogenic differentiation [275]. In this manner, hydrogels act not only as structural scaffolds but also as biophysical regulators of cell behavior [268].

However, the addition of HAp improves mechanical reinforcement and structural stability, enabling the composite to better match the load-bearing characteristics of cartilage and osteochondral tissues. The addition of HAp significantly improves mechanical stability and structural integrity, enabling better replication of the cartilage–subchondral bone interface, which is essential in osteochondral regeneration [276]. In addition, HAp particles possess a high specific surface area and strong protein adsorption capacity, enabling the adsorption and retention of adhesion proteins such as fibronectin as well as various bioactive molecules and growth factors. This property facilitates cell–material interactions and promotes integrin-mediated adhesion and downstream mechanotransduction signaling pathways that regulate stem cell behavior and tissue regeneration [202,203]. Furthermore, the incorporation of HAp into hydrogel matrices improves cellular attachment, proliferation, and extracellular matrix (ECM) production compared with hydrogel-only systems, owing to enhanced bioactivity and cell–matrix interactions [277,278].

The 3D architecture of HAp–hydrogel systems further promotes cell–cell and cell–matrix interactions, which are essential for cartilage-specific matrix production (e.g., collagen type II and glycosaminoglycans) [269].

Importantly, HAp inclusion facilitates the formation of mineral gradients, which are necessary for recreating the transition between cartilage and subchondral bone [279].

**Figure 9:**
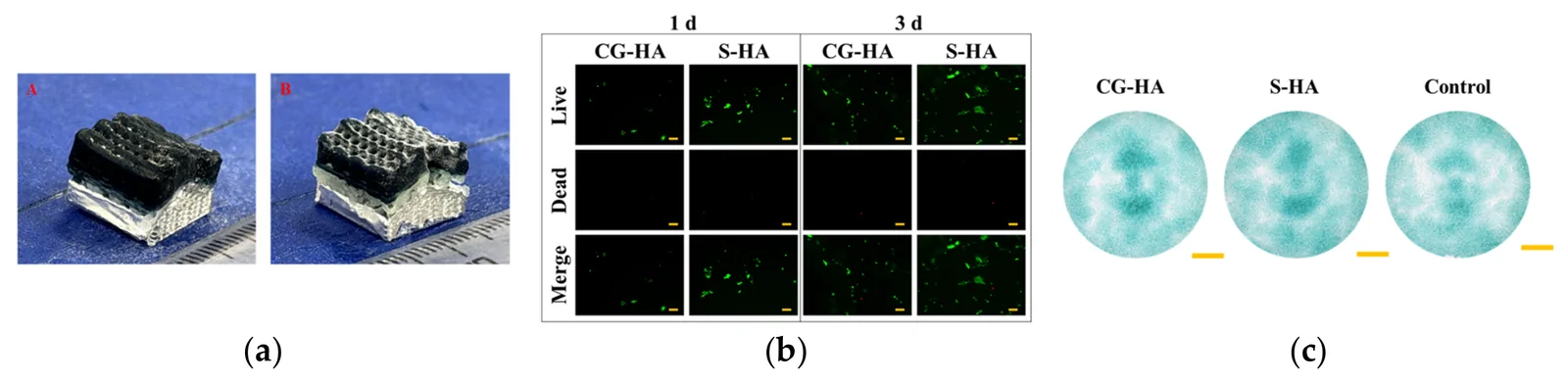
(**a**) Macroscopic morphology and HAp gradient visualization of CG-HAp and S-HAp scaffolds: (**A**) continuously graded HAp hydrogel scaffold (CG-HAp), (**B**) S-HAp; (**b**) fluorescence images showing BMSC viability on CG-HAp and S-HAp scaffolds after 1 and 3 days of culture. Live cells are shown in green and dead cells in red; scale bar = 100 μm; and (**c**) Alcian Blue staining of BMSCs cultured in extracts from CG-HAp scaffolds, S-HAp scaffolds, and blank control for 7 days (blue indicates glycosaminoglycan depositions; yellow scale bar: 3000 μm). Reproduced with permission from [280].

Recent advances in extrusion-based 3D bioprinting have enabled the precise fabrication of gradient HAp-based scaffolds that mimic the native osteochondral interface. By dynamically controlling the feeding rates of two bioinks, a continuous decrease in HAp content from bottom to top can be achieved, replicating the transition from calcified to non-calcified cartilage. These gradient scaffolds exhibit suitable shear-thinning behavior, shape fidelity, and improved compressive properties compared to stepwise designs, highlighting the mechanical advantages of continuous compositional transitions. Biologically, such constructs provide a supportive microenvironment for cell activity, promoting bone marrow mesenchymal stem cell (BMSC) adhesion and proliferation while maintaining cytocompatibility (Figure 9). Importantly, the controlled incorporation of HAp does not impair cartilage-specific ECM synthesis, indicating that spatially regulated mineralization can support both osteogenesis and chondrogenesis without compromising cartilage function [280].

**Figure 10:**
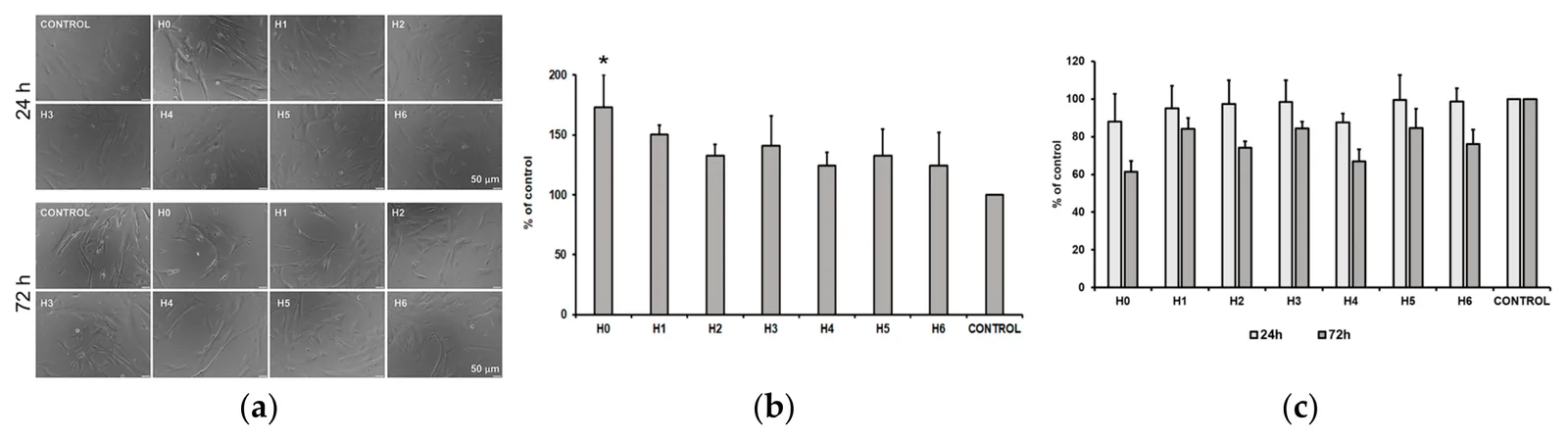
(**a**) Representative morphology images of hUC-MSCs (human umbilical cord Wharton’s jelly mesenchymal stem cells) cultured for 24 and 72 h in a particular liquid extract of chitosan-based samples (H0: chitosan (CS); H1: CS/graphene oxide (GO); H2: CS/poly(ethylene glycol) grafted graphene oxide (GO-PEG); H3: CS/reduced graphene oxide (rGO); H4: CS/GO/HAp; H5: CS/GO-PEG/HAp; H6: CS/rGO/HAp). Scale bar: 50 µm. (**b**) The cytotoxicity of liquid extracts of H1, H2, H3, H4, H5, H6 samples. (**c**) Proliferation of hUC-MSCs (human umbilical cord Wharton’s jelly mesenchymal stem cells) after 24 and 72 h of the cell culture in liquid extracts of H1, H2, H3, H4, H5, H6 samples. Data are expressed as mean ± SD; *p* < 0.05 was considered as statistically significant and labeled by (*). Reproduced with permission from [281].

In another study, gradient chitosan-based hydrogels using a freezing–gelling–thawing method, incorporating graphene derivatives (GO, rGO, GO-PEG) and HAp was developed [281]. The composite hydrogels exhibited tunable physicochemical, mechanical, and bioactive properties, with GO-PEG/HAp systems showing the most promising performance. These scaffolds demonstrated good stability, bioactivity, and cytocompatibility with human MSCs (Figure 10), highlighting their potential for tissue engineering applications.

**Figure 11:**
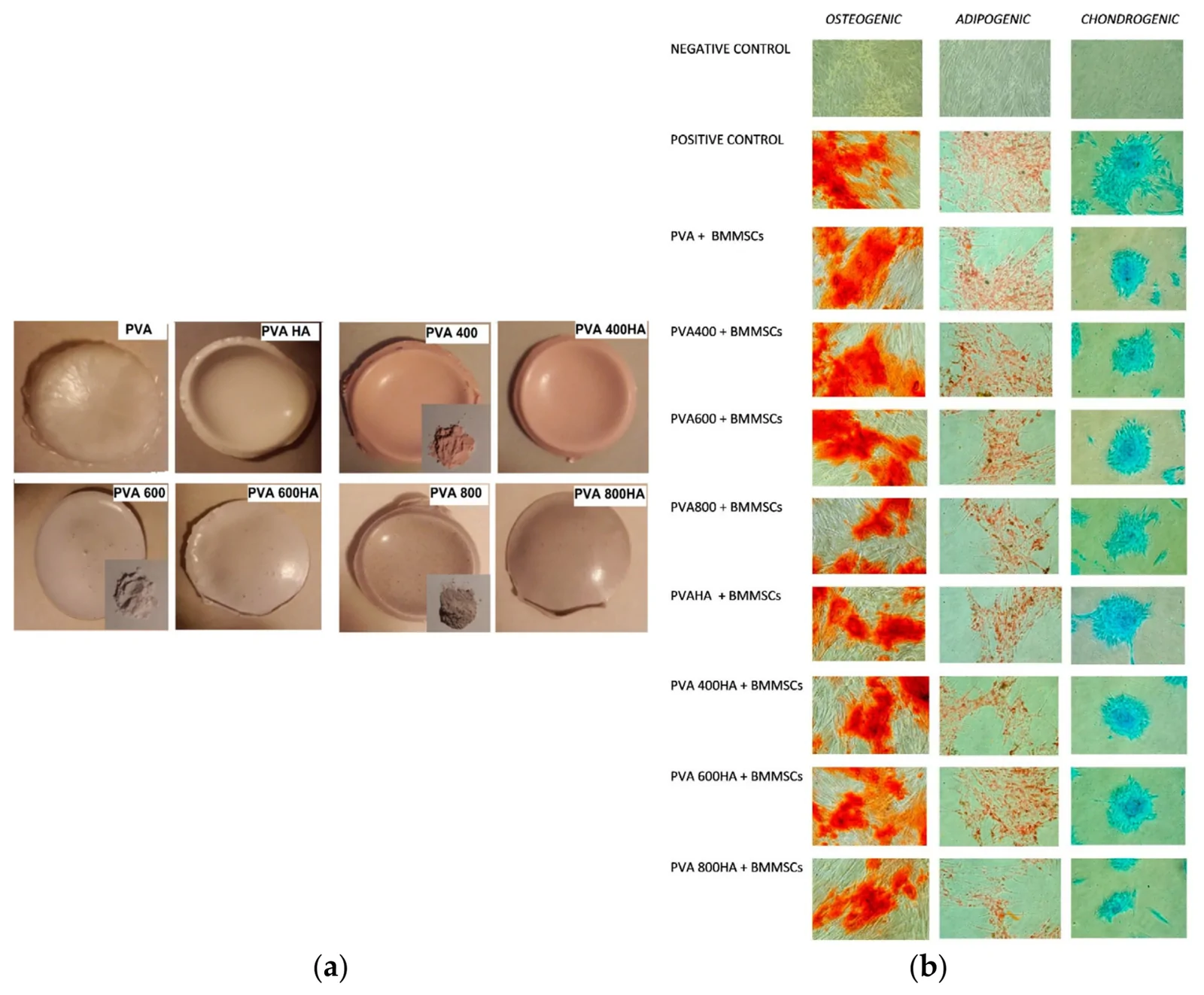
(**a**) Photographic images of poly(vinyl alcohol)/Se-doped TiO_2_ composites with and without HAp and the reference PVA specimen (10%). The inset images present the powder of Se-doped TiO_2_ particles prepared from TiO_2_ precursor after calcination at 400, 600 and 800 °C, respectively. (**b**) Differentiation potential of bone marrow mesenchymal stem cells (BMMSCs) to adipogenic, osteogenic and chondrogenic lineages, after 72 h incubation time, in the presence of PVA-based composites. Reproduced with permission from [282].

In a novel study, the authors developed PVA-based composites reinforced with Se-doped TiO_2_ nanoparticles and HAp for orthopedic applications. The composites exhibited enhanced mechanical properties and supported BMMSC viability and differentiation into osteogenic, chondrogenic, and adipogenic lineages (Figure 11). These findings highlight their potential as biomaterials for articular cartilage and subchondral bone repair [282].

Injectable hydrogel systems further extend these advantages by enabling minimally invasive administration. Their shear-thinning or in situ gelling properties allow them to (i) adapt to irregular defect geometries, (ii) ensure uniform filling and cell distribution, and (iii) reduce surgical trauma and associated complications.

These features are particularly valuable in cartilage repair, where defect shapes are often complex and difficult to treat with pre-formed scaffolds [283]. Injectable HAp–hydrogel composites extend these advantages by enabling minimally invasive delivery and in situ scaffold formation. Hydrogels ensure conformal filling of irregular defects, while HAp improves material retention and mechanical reinforcement at the defect site, enhancing long-term stability and integration [284].

At a smaller scale, hydrogel microsystems (e.g., microspheres or microgels) introduce additional functional benefits. Their high surface-area-to-volume ratio enhances (i) nutrient and oxygen diffusion, (ii) waste removal, and (iii) cell–material interactions. This improved mass transport is especially important in cartilage, where the lack of vasculature limits nutrient supply. Moreover, microscale building blocks can be assembled into modular systems, enabling spatial control over cell distribution and biochemical cues, further improving regeneration outcomes [285]. Therefore, nano-HAp plays a particularly important role due to its similarity to natural bone mineral. nano-HAp enhances (i) surface reactivity, (ii) ion exchange capacity, and (iii) cellular uptake and signaling. This results in improved MSC differentiation and tissue regeneration outcomes, especially in composite systems designed for osteochondral repair [286]. Furthermore, HAp–hydrogel composites can act as bioactive reservoirs, where hydrogels control the release of encapsulated cells or factors, and HAp enhances adsorption and sustained retention of biomolecules. This dual functionality enables spatiotemporal control of biological signals, which is critical for orchestrating cartilage regeneration processes [141].

Current evidence suggests that the most promising ECM-mimicking strategies combine the biological advantages of hydrogels with the mechanical reinforcement and bioactivity provided by HAp. Among the approaches reviewed, gradient HAp–hydrogel scaffolds appear particularly promising because they better reproduce the native osteochondral interface while supporting both chondrogenesis and subchondral bone regeneration. In addition, injectable and nanoscale HAp-containing systems offer important advantages for minimally invasive delivery, enhanced cell–material interactions, and spatial control of bioactive signals, although further optimization is still required to ensure long-term mechanical stability and clinical translation.

### 6.2. Promotion of Chondrogenic Differentiation

Although HAp is classically used in bone engineering, HAp-containing hydrogels can also regulate cartilage regeneration, especially in osteochondral defects, where successful repair depends on coordinated regeneration of cartilage, calcified cartilage, and subchondral bone [287,288]. In this context, nano-HAp serves not only as a mineral filler but also as a bioactive signaling component that influences the local cellular microenvironment through ion exchange, surface interactions, and spatial guidance of tissue formation [287,288].

At the cellular level, nano-HAp has been shown to enhance mesenchymal stem cell (MSC) adhesion, proliferation, and lineage-specific differentiation. The high surface area and bioactive nature of nano-HAp facilitate protein adsorption and integrin-mediated cell attachment, which in turn activates intracellular signaling pathways involved in cytoskeletal organization and differentiation. Experimental evidence demonstrates that HAp-containing composite hydrogels significantly improve MSC viability and promote differentiation toward osteochondral phenotypes through synergistic interactions between the hydrogel matrix and mineral phase [289,290].

Experimental osteochondral studies using GelMA/nano-HAp, alginate-nano-HAp, and collagen/HAp-based hydrogel composites show that inclusion of HAp improves both biological performance and structural integration across the cartilage–bone interface [27,49,291].

A key mechanism underlying this bioactivity is the controlled release of calcium (Ca^2+^) and phosphate (PO_4_^3−^) ions from HAp [292,293,294]. These ions serve as signaling mediators that modulate cellular responses and gene expression. In particular, Ca^2+^ activates calcium-sensitive signaling networks, including CaSR-mediated MAPK/ERK and Wnt-related pathways, which regulate MSC differentiation, extracellular matrix synthesis, and osteochondral tissue regeneration [295,296,297].

Phosphate ions contribute to matrix mineralization and cellular energy metabolism. Studies have shown that ion release from HAp-integrated hydrogels significantly enhances osteogenic differentiation and matrix synthesis even in the absence of exogenous growth factors [89]. In a nano-HAp loaded hydrogel for talar cartilage repair, nanoHAp enhanced chondrocyte proliferation, migration, and secretion of cartilage matrix components such as type II collagen and glycosaminoglycans, while also promoting features related to maintenance of the osteochondral interface [298]. Likewise, multilayer hydrogel constructs enriched with nano-HAp in the deeper zone have shown zone-specific effects on chondrogenic phenotype, with deeper mineralized regions promoting expression patterns consistent with calcified cartilage and interface maturation [299].

Another important effect of HAp is its ability to promote MSC chondrogenesis when placed in an appropriate hydrogel microenvironment. HAp alone does not universally drive a purely chondral phenotype; rather, its effect depends on particle size, concentration, scaffold architecture, and the presence of complementary cues such as decellularized cartilage matrix, gelatin methacrylate, chondroitin sulfate, or chondrogenic factors [17,300].

HAp is also important because it helps establish mineralization gradients, which are essential for reconstructing the transition from hyaline cartilage to calcified cartilage and then to subchondral bone. This transition zone is one of the hardest features to reproduce in osteochondral engineering [301,302,303,304]. For example, a high-porosity GelMA scaffold containing surface-modified nano-HAp promoted adipose-derived stromal cell differentiation and enhanced osteochondral repair in rabbit defects, indicating that uniform mineral dispersion within the hydrogel can improve both cellular signaling and tissue outcome [49]. Similarly, multilayer hydrogels combining methacrylated chondroitin sulfate, GelMA, PEGDMA, and nano-HAp recreated zonal differences in stiffness and porosity and supported spatially distinct chondrogenic responses, suggesting that nano-HAp can help reproduce the biochemical and biomechanical heterogeneity of native osteochondral tissue [299].

Gradient or bilayer hydrogel systems which include HAp in the lower or deeper phase better mimic the native interface and improve mechanical continuity between soft and hard tissues. A factorial-design study of HAp/collagen hybrid hydrogels showed that HAp content strongly affects porosity, swelling, degradation, and mechanical behavior, and that tuning mineral loading enables graded structures suitable for osteochondral regeneration [305].

In a bilayer alginate-nano-HAp/chitosan-hydrogel scaffold, the nano-HAp-containing subchondral compartment supported osteogenic differentiation while the upper hydrogel layer supported chondrogenic behavior, demonstrating how mineral gradients can direct region-specific tissue development [291].

**Figure 12:**
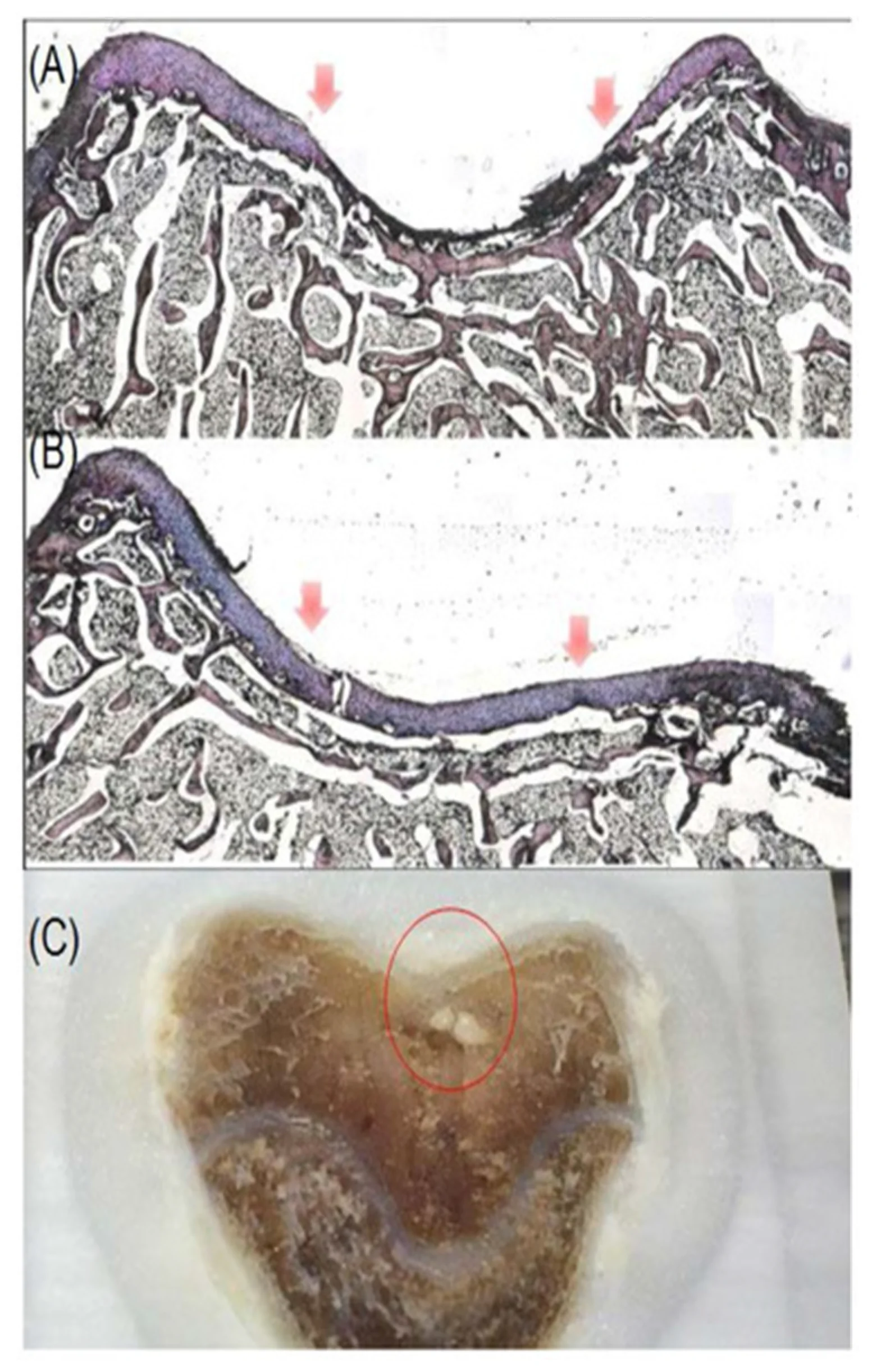
The histological staining (Alcian blue/PAS staining) and specimen of cartilage sections of rabbit after 12 weeks after implantation. (**A**) The control group with only bone formation; and (**B**) the experimental group showed bone and hyalin cartilage regeneration (arrow means defect); (**C**) The specimen of the experimental group showed good cartilage and bone regeneration on the surface layer and residual un-degraded scaffold in the deep layer. Reproduced with permission from [306].

Another study is dedicated to the fabrication of porous PCL-based scaffolds with HAp using fused deposition modeling and functionalized them with RGD peptides to enhance cell adhesion. The scaffolds were further combined with a hyaluronic acid hydrogel loaded with TGF-β1 for bioactive delivery. HAp improved mechanical strength, while the RGD-modified surface supported cell adhesion and proliferation. In vivo results showed complete osteochondral defect repair after 12 weeks (Figure 12), with formation of hyaline cartilage, highlighting the effectiveness of combining structural design with bioactive factor delivery [306].

**Figure 13:**
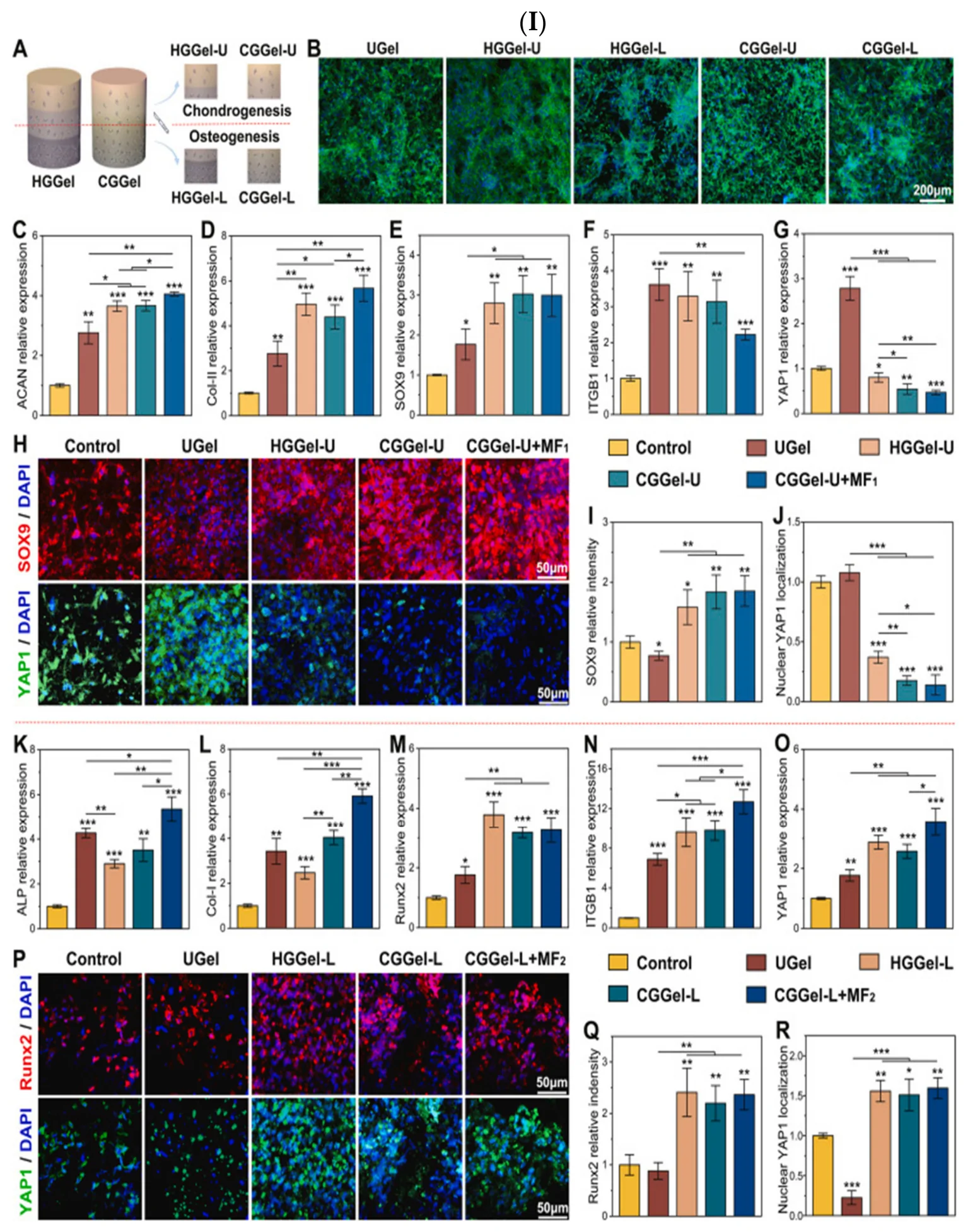

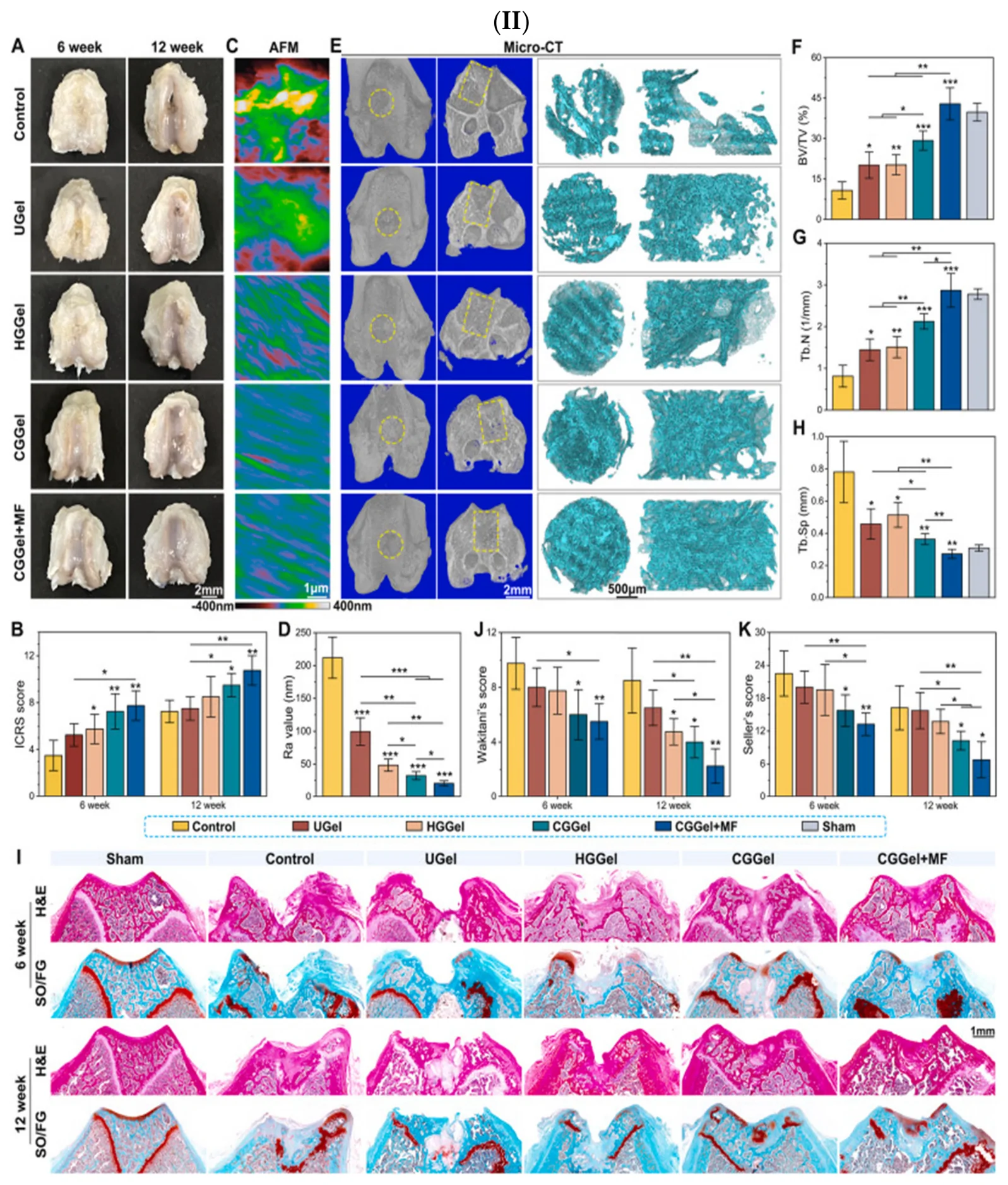
(**I**) Effect of hydrogels on BMSC chondrogenic and osteogenic differentiation: (**A**) schematic illustration of HGGel and CGGel hydrogels divided into upper (U) and lower (L) regions to induce chondrogenic and osteogenic differentiation of BMSCs, respectively; (**B**) FITC-phalloidin staining of BMSCs cultured on different hydrogels for 5 days. mRNA expression of chondrogenic markers: (**C**) ACAN, (**D**) Col-II, (**E**) SOX9, and mechanotransduction-related genes (**F**) ITGB1 and (**G**) YAP1 after 14 days; (**H**–**J**) CLSM analysis of SOX9 expression and YAP1 nuclear localization. Osteogenic differentiation assessed by mRNA expression of (**K**) ALP, (**L**) Col-I, (**M**) Runx2, together with (**N**) ITGB1 and (**O**) YAP1 after 14 days; (**P**–**R**) CLSM analysis of Runx2 expression and YAP1 nuclear localization. Data are presented as mean ± SD (* *p* < 0.05, ** *p* < 0.01, *** *p* < 0.001; n = 3). (**II**) In vivo osteochondral regeneration by gradient hydrogels: (**A**) gross appearance of regenerated osteochondral tissues in the Control, UGel, HGGel, CGGel, and CGGel + MF groups after 6 and 12 weeks, with corresponding (**B**) ICRS macroscopic scores; (**C**) surface morphology of regenerated tissues at week 12 and (**D**) quantification of surface roughness (Ra); (**E**) representative 3D micro-CT reconstructions after 12 weeks. Quantitative micro-CT analysis showing (**F**) bone volume fraction (BV/TV), (**G**) trabecular number (Tb.N), and (**H**) trabecular separation (Tb.Sp); (**I**) histological evaluation using H&E and Safranin O/Fast Green staining at 6 and 12 weeks. Histological scoring based on (**J**) Wakitani and (**K**) Sellers systems. Data are presented as mean ± SD (* *p* < 0.05, ** *p* < 0.01, *** *p* < 0.001; n = 4). Reproduced with permission from [28].

Xu et al. developed a continuous gradient hydrogel system incorporating magnetic and mechanical gradients together with spatially distributed functional metal ions [28]. The hydrogel was fabricated using poly(ethylene glycol) diacrylate/sodium alginate integrated with Fe_3_O_4_ nanoparticles coated with Mg-doped HAp (MgHAp@Fe_3_O_4_), followed by Mn^2+^-mediated secondary crosslinking. This strategy generated spatially controlled gradients in stiffness, magnetism, and bioactive ion distribution. Importantly, the gradient distribution of MgHAp@Fe_3_O_4_ and Mn^2+^ promoted region-specific differentiation of bone marrow-derived mesenchymal stem cells (BMSCs), enhancing chondrogenesis in cartilage-like regions and osteogenesis in deeper mineralized layers (Figure 13I). In vivo, the continuous gradient hydrogel achieved significant repair of full-thickness osteochondral defects in rat knee joints, demonstrating the importance of biomimetic gradient architectures for integrated osteochondral regeneration (Figure 13II).

In vivo studies provide important evidence for the translational relevance of HAp–hydrogel composites [307]. In animal models, these systems have been shown to support tissue repair by promoting new matrix deposition, improving collagen organization, enhancing vascularization, and increasing integration at the defect site [308]. Such findings are particularly valuable because they complement in vitro observations and demonstrate that the material can function in a more complex biological environment [309,310]. Overall, the animal data indicate that properly designed HA–hydrogel composites may offer meaningful regenerative advantages for cartilage, osteochondral, and bone-related applications.

Beyond ion release, HAp offers a bioactive surface for protein adsorption, which is highly relevant to regenerative signaling. Because of its surface charge and high specific surface area, nano-HAp can adsorb serum proteins, adhesion molecules, and growth factors, thereby concentrating endogenous signals at the cell–material interface. This improves cell attachment, integrin engagement, and downstream mechanotransduction, which in turn affects matrix deposition and lineage commitment [294,311,312].

In HAp-containing GelMA and collagen-based osteochondral systems, improved mineral dispersion correlated with stronger cell responses and better tissue formation, suggesting that the hydrogel matrix does not simply carry the particles but helps expose them in a biologically meaningful way [27,49].

This signaling role becomes even more evident in multifunctional hydrogel systems, where nano-HAp is combined with bioactive drugs or chondrogenic molecules. In a bilayer microsphere-scaffold composite, GelMA/nano-HAp microspheres in the lower phase provided osteoinductive and cytoprotective cues, while the upper hydrogel phase promoted chondrogenesis; together, the construct activated PI3K–AKT-mediated chondro-osteogenic signaling and improved osteochondral repair in vivo [313].

A related strategy using PVA/nano-HAp–hydrogel loaded with kartogenin enhanced tendon-bone healing and reduced cartilage degeneration after ACL reconstruction, further supporting the idea that nano-HAp can cooperate with biochemical factors to create a more regenerative microenvironment [314].

Importantly, the effect of HAp in cartilage-related hydrogels is dose- and context-dependent. Low to moderate amounts of uniformly dispersed nano-HAp can enhance cell signaling and interfacial regeneration, whereas excessive mineral loading may push cells toward hypertrophy or over-mineralization [141,315]. This is why recent successful designs emphasize surface modification of nano-HAp, controlled localization in deeper scaffold regions, or bilayer/gradient hydrogel architectures rather than homogeneous bulk mineralization throughout the entire cartilage phase [31,49].

The reviewed studies indicate that chondrogenic differentiation is optimized by combining nano-sized HAp with biomimetic hydrogel matrices that provide appropriate mechanical and biochemical cues. Rather than increasing HAp content uniformly, current evidence favors moderate HAp loading and its spatial localization within gradient or bilayer scaffolds to recreate the native osteochondral interface while minimizing undesirable hypertrophy or excessive mineralization. Multifunctional HAp–hydrogel systems incorporating bioactive molecules or controlled ion release appear particularly promising, as they synergistically enhance chondrogenesis and subchondral bone regeneration.

### 6.3. Stimulation of Angiogenesis at the Subchondral Region

Although mature articular cartilage is intrinsically avascular, angiogenesis is essential for successful regeneration of the osteochondral unit, particularly within the subchondral bone and calcified cartilage interface [316,317]. Effective vascularization ensures nutrient delivery, waste removal, and recruitment of progenitor cells, all of which are critical for long-term tissue functionality and integration [317,318].

HAp–hydrogel composites are uniquely positioned to regulate this process by combining bioactive mineral signaling with tunable hydrogel microenvironments. A key mechanism through which HAp–hydrogel systems stimulate angiogenesis is via ion-mediated signaling. The gradual release of Ca^2+^ from nano-HAp has been shown to activate angiogenic pathways, including VEGF expression and endothelial cell migration. In composite hydrogels, the polymeric network stabilizes HAp dispersion and enables sustained ion release, creating a pro-angiogenic microenvironment [295,296,297]. For instance, injectable GelMA/nano-HAp hydrogels have demonstrated enhanced vascular-related gene expression (e.g., VEGF, VEGFR-2) and endothelial activity, contributing to improved tissue regeneration outcomes [319].

Beyond direct ion effects, HAp also enhances angiogenesis through its ability to adsorb and retain pro-angiogenic growth factors, including VEGF and other cytokines. This adsorption capacity allows HAp–hydrogel composites to act as localized reservoirs, prolonging growth factor bioavailability and enabling sustained stimulation of endothelial cells. In multifunctional systems combining HAp with bioactive molecules, enhanced vascularization has been linked to improved coupling between angiogenesis and osteogenesis, a process critical for subchondral bone regeneration [313].

Importantly, angiogenesis in cartilage repair must be spatially controlled. Excessive vascular invasion into the cartilage layer can disrupt the formation of stable hyaline cartilage, whereas insufficient vascularization in the subchondral region leads to poor integration and mechanical instability [320].

HAp–hydrogel composites address this challenge through gradient and bilayer scaffold designs, where HAp is preferentially localized in deeper regions to promote vascularized bone regeneration, while the upper hydrogel layer maintains a cartilage-compatible environment. For example, bilayer hydrogel systems incorporating nano-HAp in the subchondral phase have demonstrated simultaneous osteogenic differentiation and vascularized tissue formation, alongside preservation of chondrogenic activity in the cartilage layer [291].

Advanced HAp–hydrogel systems further refine angiogenic control through stimuli-responsive and spatiotemporally regulated delivery mechanisms [303,304]. These systems can modulate the release of pro-angiogenic factors or ions in response to environmental cues (e.g., enzymatic activity, pH changes, or external stimuli), enabling precise regulation of vascularization over time [303]. Such control ensures that angiogenesis is promoted during early-stage subchondral repair but limited during later stages to preserve cartilage integrity. Recent developments in responsive hydrogel systems highlight their ability to synchronize vascularization with tissue maturation, improving overall regenerative outcomes [321].

Current evidence indicates that spatially controlled angiogenesis is more beneficial than uniform vascular stimulation. Among the strategies reviewed, gradient and bilayer HAp–hydrogel scaffolds appear the most promising because they promote vascularization within the subchondral bone while preserving the avascular environment required for stable hyaline cartilage. Responsive HAp–hydrogel systems further enhance this approach by enabling temporal regulation of angiogenic signals during different stages of osteochondral regeneration.

### 6.4. Immunomodulation and Anti-Inflammatory Effects

The inflammatory microenvironment following cartilage injury plays a decisive role in determining whether healing results in fibrotic repair or true tissue regeneration [322,323]. In osteochondral defects, excessive or prolonged inflammation leads to matrix degradation, chondrocyte apoptosis, and fibrocartilage formation, whereas a properly regulated immune response supports stem cell recruitment, differentiation, and ECM synthesis [323].

HAp–hydrogel composites have emerged as effective platforms for modulating immune responses, particularly through their influence on macrophage behavior and the broader osteoimmune microenvironment [324]. A central mechanism is the regulation of macrophage polarization. Macrophages exist along a spectrum of phenotypes, with pro-inflammatory M1 macrophages producing cytokines such as TNF-α and IL-1β that drive tissue degradation, while anti-inflammatory M2 macrophages promote tissue repair, angiogenesis, and matrix remodeling [325,326]. Studies demonstrate that hydrogel-based systems, especially when combined with bioactive components, can shift macrophage polarization from M1 toward M2, thereby establishing a pro-regenerative microenvironment [304]. For example, immunomodulatory hydrogels have been shown to significantly enhance M2 polarization and reduce inflammatory responses, leading to improved osteochondral repair outcomes [327,328].

In HAp–hydrogel composites, this immunomodulatory effect is further enhanced by the presence of nano-HAp. HAp contributes to immune regulation through ion-mediated signaling and surface interactions, which influence macrophage behavior and cytokine expression. For instance, hydrogels incorporating ion-doped nano-HAp (e.g., lithium-substituted HAp) have been shown to promote M2 polarization via activation of signaling pathways such as JAK/STAT, resulting in reduced inflammation and enhanced osteogenesis and angiogenesis [329]. Similarly, nanocomposite hydrogels containing nano-HAp have demonstrated the ability to suppress inflammatory responses while simultaneously promoting regenerative signaling, highlighting the dual role of HAp as both a structural and immunomodulatory component [289]. Importantly, immunomodulation is closely linked to osteochondral regeneration. Macrophages not only can regulate inflammation but also directly influence stem cell behavior and tissue formation [325].

Experimental studies show that M2 macrophages can enhance MSC proliferation, chondrogenic differentiation, and subchondral bone regeneration, while also reducing apoptosis and oxidative stress. In osteochondral defect models, materials that promote M2 polarization result in improved cartilage matrix deposition and more complete structural integration [330].

HAp–hydrogel systems can also modulate the immune response through spatially controlled microenvironments, particularly in gradient or bilayer scaffolds. In such designs, the HAp-rich subchondral region promotes osteogenic and immunoregulatory responses, while the upper hydrogel layer supports chondrogenesis. For example, biomimetic gradient hydrogels incorporating calcium phosphate phases have been shown to simultaneously induce M2 macrophage polarization, enhance chondrogenesis, and promote osteogenesis, enabling coordinated regeneration of cartilage and bone [331]. Furthermore, HAp–hydrogel composites can serve as delivery platforms for immunomodulatory agents, enabling precise control over inflammatory signaling. Advanced systems incorporate bioactive molecules, drugs, or extracellular vesicles that regulate macrophage behavior and cytokine expression [303]. For instance, multifunctional bilayer hydrogel systems containing nano-HAp and anti-inflammatory agents have been shown to attenuate oxidative stress, modulate macrophage polarization, and activate regenerative signaling pathways (e.g., PI3K–AKT), resulting in enhanced osteochondral repair [313]. Similarly, immunomodulated adhesive hydrogels delivering therapeutic molecules can downregulate inflammatory pathways (e.g., TNF signaling) while promoting cartilage regeneration [332]. Crucially, this controlled immunoregulation is not only about suppressing inflammation but about orchestrating a dynamic immune response [141].

Early-stage inflammation is necessary to initiate healing, but it must transition toward a regenerative phase characterized by M2 macrophage dominance, reduced pro-inflammatory cytokines, and increased production of regenerative mediators [304,325].

HAp–hydrogel composites facilitate this transition by providing sustained biochemical cues, structural support, and controlled release of signaling molecules, ultimately creating a pro-regenerative immune niche. In addition to macrophage modulation, advanced HAp–hydrogel systems have been engineered to address oxidative stress, which is another major contributor to chronic inflammation and cartilage degeneration. Reactive oxygen species (ROS) accumulation can damage cellular components, inhibit chondrogenesis, and accelerate matrix breakdown. Recent studies demonstrate that multifunctional HAp-based composite hydrogels can scavenge excessive ROS and regulate redox balance, thereby protecting cells and supporting a regenerative microenvironment [333]. This antioxidant capability further enhances the anti-inflammatory performance of the material.

The presented studies indicate that successful HAp–hydrogel composites regulate rather than simply suppress inflammation by promoting the transition from a pro-inflammatory to a regenerative immune microenvironment. Among the strategies reviewed, multifunctional and gradient HAp–hydrogel systems appear the most promising because they combine spatially controlled immunomodulation with sustained delivery of bioactive cues, thereby simultaneously supporting chondrogenesis, osteogenesis, and tissue integration.

### 6.5. Antibacterial Activity and Protection of the Regenerative Niche

Preventing infection is essential for successful cartilage repair, particularly when implanted biomaterials are used to treat focal chondral or osteochondral defects [141]. Although articular cartilage is inherently avascular and exhibits limited intrinsic healing capacity, the surrounding joint environment is highly susceptible to infection following trauma or surgical intervention [141,334]. Even low-grade bacterial contamination can severely compromise regeneration by elevating inflammatory responses, impairing cell viability, and disrupting ECM deposition [334]. Consequently, the incorporation of antibacterial functionality into HAp-based hydrogels has emerged as a critical strategy to preserve a stable regenerative niche and ensure long-term therapeutic success.

HAp-based hydrogels offer a unique platform for localized antibacterial therapy due to their high surface area, tunable porosity, and capacity to incorporate a wide range of antimicrobial agents [335]. These include metallic nanoparticles (e.g., silver), ion-doped apatites (e.g., Zn^2+^, Cu^2+^, Co^2+^), and bioactive molecules with inherent antimicrobial properties [336,337,338].

The localized delivery of such agents enables high antibacterial efficacy directly at the implantation site while minimizing systemic toxicity and reducing the risk of antibiotic resistance. For instance, an injectable hydroxypropyl methylcellulose–HAp hydrogel incorporating Ag NPs demonstrated potent antibacterial activity against both *S. aureus* and *E. coli*, while maintaining excellent biocompatibility and supporting tissue repair [339]. This dual functionality highlights the potential of HAp–hydrogels to simultaneously control infection and support regenerative processes.

In the context of cartilage and osteochondral repair, biofilm formation at the implant interface can hinder cell attachment, block nutrient diffusion, and create a chronic inflammatory microenvironment that impairs tissue regeneration [340]. Studies have demonstrated that HAp-based composite hydrogels can effectively inhibit biofilm formation through both chemical and physical mechanisms. For example, a 3D-printable carboxymethyl chitosan/gelatin hydrogel incorporating magnesium/nanodiamond dual-doped HAp exhibited strong antibacterial activity against *E. coli* and methicillin-resistant *S. aureus* (MRSA), along with significant biofilm inhibition and enhanced MSC viability [290].

Beyond direct antibacterial effects, HAp–hydrogels play a crucial role in mitigating infection-induced inflammation. Bacterial colonization triggers the release of pro-inflammatory cytokines such as tumor necrosis factor-alpha (TNF-α), interleukin-1 beta (IL-1β), and interleukin-6 (IL-6), which are known to promote cartilage degradation by upregulating matrix metalloproteinases (MMPs) and inhibiting anabolic pathways in chondrocytes [341,342,343].

By suppressing bacterial growth at an early stage, antibacterial HAp–hydrogels reduce the activation of these inflammatory cascades, thereby preserving ECM integrity and supporting chondrogenic activity. In a composite hydrogel system containing gallic acid-functionalized chitosan and polydopamine-modified HAp, effective bacterial inhibition was accompanied by enhanced osteogenic differentiation and improved tissue repair [89].

Another critical dimension of antibacterial HAp–hydrogels is the sustained and controlled release of antimicrobial agents. Unlike conventional treatments that rely on burst release or systemic administration, HAp-based systems enable prolonged delivery of antibacterial ions or molecules, maintaining effective concentrations over extended periods. This sustained activity is essential for preventing delayed infections and ensuring continuous protection during the different phases of tissue healing. Ion-substituted HAp systems, for example, can gradually release antibacterial ions while simultaneously enhancing cellular responses and bioactivity [344]. More recent studies further demonstrate that incorporating HAp into polymeric hydrogel networks improves not only antibacterial performance but also mechanical stability and cellular compatibility, which are essential for cartilage tissue engineering [345].

Current evidence suggests that multifunctional HAp–hydrogel composites incorporating antibacterial ions or nanoparticles are more effective than pristine HAp in preventing infection while maintaining cytocompatibility. Among the strategies reviewed, systems providing sustained local release of antimicrobial agents appear particularly promising, as they simultaneously prevent biofilm formation, reduce inflammation, and preserve a favorable microenvironment for cartilage and osteochondral regeneration.

### 6.6. Controlled Release of Bioactive Molecules

HAp-based hydrogels are increasingly being developed not only as structural scaffolds for defect filling, but also as multifunctional delivery systems capable of providing localized, sustained, and in some cases stimuli-responsive release of therapeutic agents [141,335,346]. This property is especially important in cartilage and osteochondral regeneration, where healing depends on the precise temporal coordination of anti-inflammatory, chondrogenic, chemotactic, and matrix-preserving signals. Hydrogel networks containing HAp are well suited because they combine the water-rich permeability of hydrogels with the adsorption capacity, ionic activity, and mineral-like surface chemistry of HAp, thereby enabling both physical entrapment and mineral-assisted binding of drugs, proteins, and signaling molecules [311].

A central advantage of HAp-containing hydrogels is that the mineral phase itself can participate in release regulation [311]. Owing to its charged surface, high specific area, and affinity for biomolecules, HAp can adsorb proteins, small molecules, and ions, reducing burst release and prolonging local bioavailability [311]. At the same time, gradual dissolution of nano-HAp can release calcium and phosphate ions, which are not merely degradation products but active biological signals that influence cell adhesion, differentiation, and matrix production [295,296,297].

In composite hydrogels, the combination of polymer network diffusion, scaffold degradation, and HAp-mediated adsorption/desorption creates a multistage release profile that is often more sustained than that of polymer-only systems [295,296,297]. This is particularly valuable in cartilage engineering, where stem or progenitor cells require prolonged exposure to instructive signals to maintain chondrogenic commitment and extracellular matrix synthesis.

Recent studies show that HAp-containing hydrogels can successfully deliver small molecules and biologically active compounds in a sustained manner. For example, an injectable nano-HAp-incorporated GelMA hydrogel was used as a delivery platform for Notoginsenoside R1, producing sustained release and significantly enhancing vascularized tissue regeneration through activation of Notch1/Akt signaling [319].

Although this study was performed in bone regeneration, the mechanism is highly relevant to osteochondral repair, where controlled pro-regenerative signaling at the subchondral interface is essential for stabilizing overlying cartilage repair. Likewise, alkyl-functionalized gellan gum hydrogels containing HAp/tricalcium phosphate nanoparticles were shown to support delivery of both dexamethasone and stromal cell-derived factor-1 (SDF-1), illustrating that apatite-containing injectable hydrogels can act as dual-delivery systems for anti-inflammatory and cell-recruiting molecules [347]. This is directly relevant to cartilage regeneration, where chemotactic recruitment and inflammation control are both necessary to support endogenous repair.

Another important strategy is the encapsulation of anti-inflammatory or chondroprotective molecules within HAp-based hydrogels for intra-articular use. A recent study described a biomimetic nano-HAp/chitosan injectable hydrogel loaded with Chikusetusaponin IVa for osteoarthritis-associated chondral injury. The system showed favorable biocompatibility, antioxidant activity, and in vivo suppression of inflammatory mediators including COX-2, iNOS, TNF-α, IL-1β, IL-6, and IL-17, together with protection against cartilage degeneration [348]. This study is especially relevant because it shows that HAp–hydrogels can be used not only for hard-tissue applications, but also as intra-articular drug depots that preserve the cartilage microenvironment by sustained local release of anti-inflammatory bioactives.

Beyond conventional sustained release, recent studies have demonstrated that HAp hydrogels can be engineered to respond to environmental cues such as pH, temperature, and near-infrared (NIR) irradiation, allowing more precise spatiotemporal control over therapeutic delivery. One of the strongest examples is the Ce/MnHAp-containing injectable hydrogel reported for osteochondral defect repair, in which the HAp component was integrated into a bilayer system with dual responsiveness to pH and NIR stimulation. Under these conditions, the hydrogel enabled controlled release of Ce/MnHAp particles and bioactive ions, while also modulating reactive oxygen species and promoting M2 macrophage polarization; in a rabbit osteochondral defect model, this system significantly accelerated regeneration [349].

The significance of pH-responsive release in osteochondral repair lies in the fact that degenerative or inflamed joints often exhibit altered local acidity. A mildly acidic microenvironment can arise in inflamed cartilage and subchondral lesions due to high metabolic activity, hypoxia, and inflammatory cell infiltration. Hydrogels designed to alter release behavior under acidic conditions can therefore provide on-site therapeutic adaptation, delivering bioactive ions or drugs more efficiently where pathology is most active. In the study by Heng et al., the pH-sensitive release behavior of the Ce/MnHAp hydrogel enabled adaptive delivery under the pathological microenvironment, which is highly relevant to osteoarthritis and osteochondral injury [349].

Temperature-responsive behavior is also important in injectable HAp–hydrogel systems, especially for minimally invasive administration. Thermosensitive hydrogels can remain fluid during injection and gel in situ at physiological temperature, thereby improving defect filling, retention, and local residence time of loaded therapeutics. Although not every thermoresponsive cartilage hydrogel contains HAp, this design principle is increasingly being merged with mineralized systems for bone-cartilage applications. Injectable HAp-containing scaffolds with thermotropic or in situ gelation behavior have been described as platforms for cell and biomolecule release, including the gellan gum/HAp-TCP system that preserved injectability while enabling cargo delivery [347]. In addition, broader work on smart injectable hydrogels highlights the therapeutic importance of pH-, temperature-, and ion-responsive release behavior for cartilage and bone repair, even though these papers are reviews rather than primary studies [350,351].

Another important dimension of controlled release is sequential or stage-dependent delivery. In cartilage repair, the earliest phase often requires inflammation control and cell recruitment, while later stages require chondrogenic induction and matrix maturation. Although not HAp-based, a notable cartilage regeneration study showed that a hydrogel with stage-dependent release of tannic acid and kartogenin could first alleviate inflammation and oxidative stress, then induce chondrogenic differentiation, ultimately supporting full-thickness cartilage regeneration [352].

Taken together, above presented studies collectively suggest that multifunctional HAp–hydrogel systems integrating sustained and stimuli-responsive release represent the most promising delivery strategy for osteochondral regeneration. By combining controlled release of therapeutic molecules with the intrinsic bioactivity of HAp, these systems can coordinate inflammation control, cell recruitment, and chondrogenic differentiation more effectively than conventional passive delivery platforms.

## 7. From Material Design to Clinical Translation

HAp–hydrogel composites developed for cartilage and osteochondral regeneration remain largely within the experimental stage. In this respect, recent investigations of such systems have concentrated on scaffold fabrication and physicochemical characterization, spatial control of mineral or therapeutic-ion distributions, in vitro chondrogenic and osteogenic responses, and proof-of-concept evaluation in small-animal defect models [27,28,280,349].

Among recent studies, a construct combining an HAp–collagen scaffold with a thermoresponsive PLGA–PEG–PLGA thermogel and bone-marrow-derived mesenchymal stem cells was assessed in a rabbit osteochondral-defect model. The treatment promoted osteochondral repair, particularly when combined with low-frequency electromagnetic stimulation, but the study did not address large-scale manufacture, or clinical testing [27].

Further evidence comes from recently developed mineralized hydrogel systems evaluated in small-animal osteochondral-defect models. Xu et al. produced a continuous-gradient PEGDA–alginate hydrogel containing Mg-doped HAp–Fe_3_O_4_ particles and an opposing Mn^2+^ gradient. The construct promoted regionally distinct chondrogenic and osteogenic differentiation of bone-marrow-derived mesenchymal stem cells and improved repair of full-thickness osteochondral defects in rat knee joints; however, the investigation remained focused on laboratory fabrication, mechanistic analysis, and short-term preclinical performance rather than manufacturing scale-up or clinical translation [28].

Similarly, Heng et al. developed an injectable HAp-containing hydrogel with nanozyme activity and mild photothermal responsiveness. In a rabbit osteochondral-defect model, the treatment modulated the local inflammatory environment and enhanced cartilage and subchondral-bone repair over 12 weeks, but no evidence of standardized production, regulatory assessment, or human testing was reported [349].

More recently, Wang et al. [280] developed a curved GelMA–alginate hydrogel scaffold with a continuous HAp gradient using dual-channel extrusion-based 3D printing. By varying the relative flow rates of two bioinks, the authors reproduced the gradual decrease in mineral content from the calcified cartilage-facing region toward the non-calcified cartilage zone. Compared with a step-gradient construct, the continuously graded scaffold showed a smoother compositional transition, stable swelling and degradation behavior, and improved compressive performance. It also supported bone-marrow-derived mesenchymal stem-cell adhesion and proliferation in vitro. Importantly, extract-based assays indicated that neither HAp incorporation nor its graded distribution impaired the synthesis of cartilage-associated extracellular-matrix components. Nevertheless, the work remained limited to material characterization and in vitro biological assessment. The study therefore represents a useful advance in the fabrication of biomimetic cartilage scaffolds, while also illustrating the additional validation still required before such constructs can progress toward clinical translation.

It is important to emphasize that all the reported findings represent important steps toward clinically usable composite systems, particularly because they address several material attributes that would ultimately influence injectability, biological safety, and local therapeutic performance. Nevertheless, the available evidence is still dominated by physicochemical characterization and cell-based testing, with limited validation in clinically relevant cartilage or osteochondral models and no demonstration of routine clinical evaluation. HAp–hydrogel technology should therefore be regarded as an actively progressing preclinical platform: its translational potential is increasingly well supported at the material level, but further in vivo validation, standardized manufacturing, stability assessment, sterilization studies, and regulatory-quality testing will be required before clinical application in cartilage regeneration can be considered realistic.

### 7.1. Preclinical-to-Clinical Model Translation

One of the main translational challenges is the limited extent to which early experimental models reproduce the biological and mechanical conditions of the human joint. HAp–hydrogel constructs have produced encouraging cartilage and osteochondral repair in rodent and rabbit defects. Such small-animal experiments remain valuable for establishing initial safety, biocompatibility, and regenerative potential; nevertheless, they provide only partial insight into how an implant may perform within a continuously loaded and potentially inflammatory human articulation. More recently, the translational relevance of HAp-based osteochondral scaffolds has been strengthened by successful evaluation in sheep models, including multilayer collagen/nano-HAp, collagen/magnesium-substituted HAp, and other biomimetic scaffold designs, which demonstrated improved cartilage repair, subchondral bone regeneration, and long-term tissue integration under clinically relevant loading conditions [353,354,355,356]. Despite these encouraging findings, the current preclinical evidence base remains limited, as relatively few studies have progressed to large-animal models and no HAp–hydrogel composite systems for cartilage or osteochondral regeneration have yet entered clinical trials. This limited translational evidence represents a major barrier to clinical adoption.

From the current body of evidence, the most consistent and clinically translatable HAp–hydrogel composite designs are biomimetic biphasic or gradient constructs that reproduce the structural and compositional heterogeneity of the osteochondral unit. These systems generally combine a cartilage-mimicking hydrogel phase with a mineralized HAp-containing layer, thereby supporting chondrogenesis in the superficial region while simultaneously promoting subchondral bone regeneration and stable osteochondral integration [302,357,358]. Across multiple rabbit and rat osteochondral defect models, such constructs have demonstrated reproducible improvements in hyaline-like cartilage formation, collagen type II deposition, glycosaminoglycan accumulation, and coordinated subchondral bone remodeling compared with homogeneous scaffolds or hydrogel-only controls [291,359]. Moreover, advances in three-dimensional printing and gradient manufacturing have enabled increasingly precise spatial distribution of HAp within hydrogel matrices, improving mechanical compatibility with native osteochondral tissue while preserving injectability or defect conformity where required [141,359]. Conversely, the least mature approaches are highly multifunctional HAp–hydrogel platforms that integrate multiple therapeutic components, including stem cells, growth factors, magnetic nanoparticles, enzyme-responsive systems, or immunomodulatory agents. Although these multifunctional composites frequently report superior biological outcomes in small-animal studies, the relative contribution of each functional component remains difficult to distinguish, and manufacturing complexity substantially increases translational challenges [141]. An additional consideration concerns the role of HAp itself. While HAp significantly enhances osteoconductivity, reinforces the mechanical properties of hydrogel matrices, and promotes regeneration of the subchondral bone compartment, excessive HAp incorporation or poorly controlled particle distribution may alter hydrogel viscoelasticity and potentially impair the formation of stable hyaline cartilage if the mineral phase extends into the superficial cartilage region. Recent studies therefore increasingly emphasize controlled HAp gradients or spatial compartmentalization, rather than homogeneous mineral dispersion, as a more physiologically relevant strategy for osteochondral regeneration [291,359].

Progress toward clinical testing of HAp–hydrogel implants should therefore move beyond evidence of defect filling and include rigorous evaluation of safety, tissue integration, matrix composition, and functional performance in clinically relevant large-animal models [360].

### 7.2. Manufacturing and Reproducibility

Manufacturing reproducibility remains an important consideration in the development of HAp–hydrogel composites. The printability and final properties of HAp-reinforced constructs were shown to be closely influenced by the composition of the polymer matrix, the amount and characteristics of the mineral phase, ink viscosity, processing temperature, deposition conditions, and the selected crosslinking or post-treatment procedure [361]. Variations in these parameters may affect filament formation, shape retention, pore architecture, degradation, and mechanical performance. Additive-manufacturing approaches can improve control over scaffold geometry, pore size, and interconnectivity compared with several conventional fabrication methods, and some HAp-containing formulations have produced regular structures with reproducible macroporosity and satisfactory bonding between deposited layers. Future HAp–hydrogel products will therefore require clearly defined raw-material attributes, controlled processing conditions, and quantitative acceptance criteria to ensure that independently produced constructs retain comparable structural and functional properties [361].

### 7.3. Regulatory and Standardization Barriers

Regulatory development is particularly demanding for hydrogel-based cartilage products because their composition, biological activity, and intended mode of use can vary considerably. Karami et al. distinguish between cell-based and non-cell-based constructs and emphasize that the applicable regulatory pathway depends on the characteristics and risk profile of the final product [362]. An acellular HAp–hydrogel scaffold designed primarily as an implantable support may be assessed within a medical-device framework, whereas the incorporation of viable cells or other biologically active components is likely to introduce additional regulatory and characterization requirements. The need to control starting-material quality, manufacturing reproducibility, degradation, biocompatibility, mechanical and rheological performance, implantation-related risks, and sterilization was also highlighted [362]. However, regulatory authorities may not provide detailed testing specifications for every hydrogel device. For this reason, the clinical target, product specifications, quality system, design controls, and risk-management strategy should be established early in development, preferably before extensive preclinical testing begins [362].

### 7.4. Integration and Long-Term Stability

Long-term integration across the osteochondral unit remains an important translational challenge. An effective construct must support cartilage regeneration at the articular surface while maintaining continuity with the calcified-cartilage region and the underlying subchondral bone. These compartments differ substantially in cellular organization, extracellular-matrix composition, porosity, mineralization, and mechanical properties, making a uniform scaffold unlikely to provide suitable conditions throughout the entire defect. HAp and related calcium phosphate phases can help reproduce the mineral-rich and mechanically stiffer environment of the bone-facing region, but their amount and spatial distribution must be carefully controlled, as excessive mineralization may reduce hydrogel hydrophilicity and biological activity. Degradation kinetics are similarly important: if the cartilage- and bone-oriented regions degrade at different rates, the intended gradient may be lost and interfacial failure may occur under cyclic loading. Gradient and multilayered hydrogels offer a rational means of coordinating these regional requirements, although their long-term durability, interfacial stability, and functional remodeling still require more extensive evaluation in clinically relevant models [363].

### 7.5. Immunogenicity and Biocompatibility

Biocompatibility should be evaluated using the complete HAp–hydrogel formulation rather than inferred solely from the established use of its individual constituents. The biological response may depend on polymer variability or impurities, residual monomers, crosslinking-related chemicals, and degradation products. These formulation-related factors may contribute to cytotoxicity, inflammation, immunogenicity, delayed foreign-body reactions, fibrosis, or impaired cartilage regeneration [364]. Functional modification of the mineral phase may further change the biological behavior of the composite. In an injectable bilayer hydrogel developed for osteochondral repair, cerium- and manganese-modified HAp provided nanozyme and stimulus-responsive functions, contributed to the regulation of excessive reactive oxygen species, promoted M2-associated macrophage polarization, and improved osteochondral-defect repair under mild photothermal treatment [349]. Together, these findings indicate that both the base formulation and any added bioactive or responsive components should be included in the overall biocompatibility assessment. Accordingly, short-term cytocompatibility assays should be complemented by longer-term evaluation of local inflammatory and foreign-body responses, along with potential systemic effects [364].

### 7.6. Translation of Functional Outcomes

Translational evaluation should extend beyond histological appearance and incorporate structural, biomechanical, and clinically relevant functional outcomes. Preclinical studies of emerging hydrogel and scaffold systems frequently assess extracellular-matrix deposition, cartilage-related marker expression, defect repair, and subchondral-bone regeneration, often in short-term experiments or small-animal models. Although such measures are valuable during early development, they do not by themselves establish durable biomechanical restoration or sustained clinical benefit. A more clinically informative assessment should combine tissue quality and integration with mechanical performance under physiological loading, pain and functional outcomes, and evidence of long-term joint preservation. Extended follow-up is particularly important because favorable early findings may not persist under prolonged loading, while tissue stability, integration, and durability can only be established over time. More broadly, the limited predictive relationship between preclinical performance and durable clinical benefit, together with the absence of harmonized structural, biomechanical, and patient-centered endpoints, remains an important barrier to clinical translation [365].

### 7.7. Sterilization Compatibility

Sterilization compatibility has received limited systematic attention in HAp–hydrogel research for cartilage and osteochondral repair, being described primarily as a preparatory step before biological testing [220]. Current evidence therefore does not identify a validated sterilization method that reliably preserves the cartilage-relevant properties of the complete HAp–hydrogel composite. Addressing this gap will require direct comparison of clinically applicable sterilization procedures, followed by post-treatment evaluation of physicochemical stability, mechanical and tribological performance, cytocompatibility, and storage behavior.

### 7.8. Cost-Effectiveness and Scalability

A clinically translatable HAp–hydrogel system must not only perform effectively but also be amenable to reproducible and cost-conscious manufacture. Although complex formulations and extensive material modification may improve selected experimental properties, they can also introduce additional requirements for process control, impurity analysis, safety evaluation, and quality assurance. These burdens may lengthen production, increase costs, and make consistent product quality more difficult to maintain during scale-up. Cell-based products can entail further procedural and manufacturing constraints, particularly when terminal sterilization is not feasible and tightly controlled production conditions are required. Acellular composites likewise need to be compatible with scalable fabrication, validated sterilization, suitable packaging and transportation, adequate shelf life, and straightforward surgical handling. Future development should therefore balance material complexity and functional performance against reproducibility, storage stability, process simplicity, clinical usability, and the magnitude of the expected therapeutic benefit. If these considerations are addressed only after laboratory optimization, even technically promising HAp–hydrogel formulations may encounter substantial barriers to manufacture and clinical implementation [366].

## 8. Conclusions and Future Perspectives

This study provides a critical overview of recent advances in the design and application of hydroxyapatite (HAp)–hydrogel composites for cartilage and osteochondral regeneration, with particular emphasis on material design strategies, mechanisms of biological activity, and current translational challenges.

HAp–hydrogel composites represent a promising class of biomaterials for cartilage and osteochondral regeneration, combining the hydrated, cell-supportive environment provided by hydrogels with the bioactive and mechanically reinforcing properties of HAp. Their versatility lies in the ability to tailor physicochemical and biological characteristics to support cell viability, modulate inflammatory responses, enable the controlled delivery of therapeutic agents, and more closely reproduce the structural complexity of native osteochondral tissues. Despite these advantages, several challenges continue to limit their clinical translation. In particular, achieving an appropriate balance between mechanical stability, degradation kinetics, injectability, and long-term biocompatibility remains a critical objective in the design of next-generation composite systems. Addressing these limitations will require continued advances in material engineering, fabrication methodologies, and functional composite design. Equally important is the generation of robust preclinical and translational evidence capable of demonstrating consistent regenerative outcomes in clinically relevant models. Continued progress in these areas is expected to further strengthen the potential of HAp–hydrogel composites as effective platforms for osteochondral tissue regeneration and repair.

## Figures and Tables

**Table 1:** Principal dopant species and their preferential substitution sites within the apatite lattice, together with the intended biological function and the representative mechanistic rationale.

Dopant/Substitution Site	Intended Biofunction	Representative Biofunctional Rationale or Effect	Ref.
Sr^2+^ → Ca^2+^ sites	Osteogenesis and angiogenesis	Sr substitution in HAp improves the physicochemical properties of HAp and enhances osteogenic differentiation, bone regeneration, and pro-angiogenic activity through increased VEGF/BFGF-associated responses	[51]
Mg^2+^ → Ca^2+^ sites	Promotion of osteogenesis and implant osseointegration	Mg incorporation into HAp influences crystal size, strength, and structural integrity, while promoting osteoblast and fibroblast proliferation, enhancing mineralization, and improving osseointegration of implants	[32]
Zn^2+^ → Ca^2+^ sites	Antibacterial activity and support of osteogenic differentiation	Zn-HAp inhibited the growth of multiple bacterial strains and Candida albicans, while maintaining mesenchymal stem cell viability and supporting osteogenic differentiation. Zn incorporation was also reported to stimulate osteoblastic activity and enhance osteogenesis	[52]
Ag^+^ → Ca^2+^ sites	Antimicrobial and antifungal activity with preservation of biocompatibility	Ag-HAp thin films exhibited broad antimicrobial and antifungal activity, inhibited Candida albicans biofilm formation, and maintained compatibility with MG63 osteoblast-like cells. The coatings were proposed as promising materials for biomedical implant coatings intended to reduce post-surgical infections while supporting cell adhesion and proliferation	[53]
SiO_4_^4−^ → Si incorporated into HAp NPs	Enhancement of scaffold bioactivity and osteogenic differentiation	PCL/Si-HAp composite films supported MC3T3-E1 adhesion and growth and enhanced osteogenic differentiation compared with PCL/HAp films, as indicated by increased ALP activity and greater extracellular matrix mineralization/calcium content	[54]
SeO_3_^2−^/SeO_4_^2−^ → PO_4_^3−^ sites	Antibacterial activity, antioxidant, antimicrobial, enhanced bioactivity, cytocompatible response	Se-Sr co-substituted HAp showed antibacterial activity against both Gram-negative *E. coli* and Gram-positive *S. carnosus*. Sr co-substitution was used to offset the cytotoxic effect of Se and to improve cell viability and bioactivity. Selenium substitution provided antimicrobial and antioxidant-related effects while maintaining cytocompatibility and enhanced biological activity	[55,56]
Cu^2+^ → Ca^2+^ sites	Antibacterial and osteogenic activity	Copper substitution imparted antibacterial activity and was discussed as beneficial for bone formation and biomedical applications	[56]
Mn^2+^ → Ca^2+^ sites	Osteogenic and antibacterial activity	Mn-HAp exhibited predominant rod-like morphology, antibacterial activity, strong hemocompatibility, and enhanced osteoblast adhesion, proliferation, and cytocompatibility	[57]
Co^2+^ → Ca^2+^ sites	Corrosion resistance, adhesion strength, and biocompatibility	Co^2+^-HAp coatings on Ti6Al4V improved corrosion resistance, adhesion strength, and biocompatibility	[58]
Ce^3+^/Ce^4+^ → Ca^2+^ sites	Antimicrobial coating functionality with osteoconductive relevance	Ce-HAp coatings contained Ce as a mixture of Ce^3+^ and Ce^4+^ ions substituting Ca^2+^ sites. Both 5Ce-HAp suspensions and coatings inhibited CFU development for all tested microbial strains	[59]
Fe^2+^/Fe^3+^ → Ca^2+^ sites	Magnetic responsiveness and imaging	Fe-HAp exhibits intrinsic superparamagnetic behavior, enabling magnetic-field responsiveness, MRI contrast capability, and hyperthermia-mediated therapeutic applications	[60]

**Table 2:** Key functional parameters of hydrogels for healing applications, including their biological roles, design handles, and supporting literature.

Property	Role in Healing Applications	Design Handles	Ref.
Swelling behavior	Maintains moist environment, absorbs exudate, permits nutrient/oxygen and drug transport; excessive swelling can compress tissue	Crosslink density, polymer hydrophilicity, charge, pore size, incorporation of hydrophobic segments	[166,167,168]
Mechanical strength	Provides structural support, protects tissue, transmits appropriate mechanical cues to cells, matches local tissue stiffness	Crosslink type/density, double/multinetworks, nanofillers/fibers, mechanical gradients, interpenetrating networks	[169,170,171]
Degradation kinetics	Matches scaffold persistence to repair timeline; provides space for neotissue, avoids long-term barrier to regeneration	Labile linkages (ester, acetal, enzymatically cleavable), oxidation degree, polymer composition, network density, enzyme-sensitive motifs	[172,173,174,175,176]
Injectability & self-healing	Enables minimally invasive delivery, conformal filling of irregular defects, 3D printing; self-healing maintains integrity under motion and cyclic loading	Dynamic covalent bonds, supramolecular interactions, shear-thinning networks, in situ/photo-crosslinking, reversible physical gels	[166,177,178]
Biocompatibility	Ensures safety (low toxicity, acceptable immune response) and supports cell adhesion, proliferation, angiogenesis, and pro-regenerative immune polarization	Polymer source (natural vs. synthetic), purity, endotoxin removal, degradation products, surface chemistry/ligands (e.g., RGD), stiffness window	[168,179,180,181,182]

**Table 3:** Comparative design features of HAp–hydrogel composites for cartilage and osteochondral repair.

Incorporation Route	HAp Scale	Main Advantage	Main Limitation	Ref.
Physical blending	Nano or micro	Simple processing and easy formulation	Particle aggregation at higher loading	[228]
In situ mineralization	Mostly nano	Better dispersion and stronger interface	More complex chemistry and control	[241]
Surface-functionalized HAp	Nano rods/fibers	Higher modulus and bioactivity	Brittleness if overloaded	[228]
Core–shell or hierarchical systems	Mixed	Separate load-bearing and release functions	Fabrication complexity	[242]
Stimuli-responsive systems	Nano or hybrid	Adaptive release and remodeling	Multivariable optimization needed	[243]

**Table 4:** Comparison of the main fabrication strategies used for HAp–hydrogel composites, highlighting their principles, advantages, limitations, mechanical characteristics, and suitable biomedical applications.

Fabrication Strategy	Principle	Advantages	Limitations	Mechanical Characteristics	Suitable Biomedical Applications
Freeze-drying/Lyophilization	Aqueous HAp–polymer suspensions are frozen and the ice crystals removed by sublimation under vacuum, leaving an interconnected macroporous scaffold whose architecture is dictated by freezing rate and ice-crystal growth direction [224]	Solvent-free, simple and scalable; yields highly porous (70–95%) scaffolds with tunable, open interconnected porosity that favors cell infiltration and nutrient diffusion [247,248]; compatible with many natural polymers (collagen, agarose, alginate) and HAp loadings [249]	Slow processing (freezing plus extended sublimation cycles); limited control over pore-shape uniformity; scaffolds are mechanically weak/brittle in the dry state and often require post-crosslinking to withstand physiological loads [248]	Compressive moduli typically in the low-kPa to few-MPa range depending on HAp content and network density; bilayered freeze-dried constructs (e.g., agarose–HAp/alginate–HAp) reproduce a graded stiffness mimicking the cartilage-to-bone transition [249]	Osteochondral and cartilage–bone interface scaffolds, where a bilayered porous architecture supports zonal differentiation of chondrocytes and osteoblasts [224,249]; growth-factor-loaded bone scaffolds (e.g., BMP-2) [248]
In situ biomimetic mineralization	HAp nanocrystals are nucleated and grown directly within a pre-formed hydrogel network—often functionalized with acidic/anionic groups mimicking non-collagenous bone proteins—via alternating or simultaneous exposure to calcium and phosphate precursor solutions [250]	Produces intimate, nanoscale HAp–polymer integration that closely mimics natural ECM mineralization, improving interfacial bonding and bioactivity versus physically blended composites [250]; mineral content/distribution tunable by cycle number and precursor concentration [250]	Multi-step, time-consuming mineralization cycles; achieving mineral homogeneity through thick or dense hydrogels is difficult, and uncontrolled crystal growth can compromise hydrogel transparency and elasticity [250]	Progressive stiffening with increasing mineralization cycles; storage/compressive moduli rise measurably with HAp content while retaining hydrogel viscoelasticity and self-healing behavior in graft-copolymer systems [251,252]	Bone regeneration scaffolds with enhanced osteoconductivity and biocompatibility [250]; demineralized-tissue interfaces relevant to osteochondral repair [250]
3D printing/bioprinting	HAp particles or nanofibers are dispersed within a printable ink (e.g., alginate, gelatin, alginate–gelatin) and deposited layer-by-layer via extrusion or inkjet printing, followed by physical or ionic crosslinking to fix the printed architecture [253,254]	Precise, patient-specific control over scaffold geometry, pore architecture and spatial HAp distribution; supports gradient/zonal designs suited to cartilage’s layered structure and allows co-printing with cells for direct bioprinting [17,255]	Printability (viscosity, shear-thinning behavior) must be balanced against mechanical performance; HAp loading above certain thresholds can clog nozzles or reduce print fidelity, and post-print crosslinking uniformity remains challenging [254]	Compressive modulus and printing fidelity increase with HAp content up to an optimum, beyond which brittleness or reduced resolution occurs; crosslinking dynamics (e.g., calcium-ion diffusion in alginate–HAp bioinks) govern final stiffness and shape retention [254]	Patient-specific cartilage and osteochondral scaffolds; cartilage-mimetic constructs for defect repair; cell-laden bioprinted grafts for articular cartilage regenerative engineering [253]
Freeze–thaw physical crosslinking	Aqueous polymer solutions (typically PVA or PVA/PVP) loaded with HAp particles undergo repeated freeze–thaw cycles that induce crystallite formation and physical (non-covalent) crosslinking of polymer chains, entrapping HAp within the network without chemical crosslinkers [220]	Solvent- and crosslinker-free process yielding elastic, cartilage-like hydrogels with high water content and biocompatibility; the number of freeze–thaw cycles offers a simple lever to tune stiffness and degradation rate [220,256]	Mechanical properties are strongly cycle-number- and composition-dependent, with batch-to-batch variability; physical crosslinks are weaker than covalent networks and may loosen under prolonged hydration or cyclic loading [257,258]	PVA/PVP–HAp hydrogels display rubber-like elasticity, with compressive/tensile moduli approaching native-cartilage ranges after optimized cycling; HAp reinforcement measurably improves stiffness and wear resistance versus HAp-free controls [220,256,258]	Cartilage replacement and load-bearing soft-tissue substitutes—reported specifically for cartilage applications—as well as bone-adjacent composite scaffolds [220,257]
Ionic/chemical crosslinking (alginate-based)	Alginate (or another polyanionic polysaccharide) chains are crosslinked through divalent/trivalent cation exchange (Ca^2+^, Sr^2+^, Ba^2+^) with backbone carboxylate groups, or through covalent chemical crosslinkers, entrapping HAp nanoparticles within the stabilized network [259,260,261]	Mild, cytocompatible gelation at room temperature without organic solvents; choice of crosslinking cation (e.g., Sr^2+^, Ba^2+^ instead of Ca^2+^) can simultaneously deliver osteogenic/therapeutic ions while forming the gel [259]	Ionically crosslinked alginate–HAp gels are prone to swelling, cation exchange with physiological fluids, and gradual mechanical softening/degradation unless combined with a secondary (dual) crosslinking strategy [260,262]	Compressive strength and stiffness increase with HAp loading and crosslinking density; dual-crosslinked (ionic + secondary) aerogel/hydrogel scaffolds show markedly improved mechanical stability over single-step ionically gelled controls [261,262]	Injectable or moldable bone and osteochondral scaffolds; platforms combining ion-delivery/osteogenic signaling with structural support [259,261]
Electrospinning	A polymer solution containing dispersed HAp nanoparticles (or a coaxial/emulsion setup) is drawn into ultrafine fibers under a high-voltage electric field, producing non-woven nanofibrous mats that mimic the fibrillar architecture of native extracellular matrix [263,264,265]	High surface-area-to-volume nanofibrous scaffolds with ECM-mimetic topography promoting cell attachment and guided tissue ingrowth; coaxial/emulsion configurations allow core–shell control over HAp placement and controlled release of bioactive agents [263,264]	Uniform HAp dispersion within fine fibers is difficult, and HAp agglomeration can disrupt spinning or create defect sites; resulting mats are thin, with lower bulk mechanical strength than bulk hydrogels, limiting standalone load-bearing use [265,266]	Tensile strength and modulus of HAp/polymer nanofiber mats increase with HAp incorporation up to an optimal loading, beyond which fiber-diameter uniformity and mechanical continuity decline [265,266]	Nanofibrous scaffolds for guided bone and soft–hard tissue-interface regeneration; reinforcing/surface layers combined with bulk hydrogels for composite cartilage–bone constructs [263,266]

## Data Availability

No new data were created or analyzed in this study. Data sharing is not applicable to this article.
